# A Proteome Translocation Response to Complex Desert Stress Environments in Perennial *Phragmites* Sympatric Ecotypes with Contrasting Water Availability

**DOI:** 10.3389/fpls.2017.00511

**Published:** 2017-04-13

**Authors:** Li Li, Xiaodan Chen, Lu Shi, Chuanjing Wang, Bing Fu, Tianhang Qiu, Suxia Cui

**Affiliations:** College of Life Sciences, Capital Normal UniversityBeijing, China

**Keywords:** *Phragmites*, desert-dune ecotype, combined stresses, DIGE, proteome translocation, membrane association

## Abstract

After a long-term adaptation to desert environment, the perennial aquatic plant *Phragmites communis* has evolved a desert-dune ecotype. The desert-dune ecotype (DR) of *Phragmites communis* showed significant differences in water activity and protein distribution compared to its sympatric swamp ecotype (SR). Many proteins that were located in the soluble fraction of SR translocated to the insoluble fraction of DR, suggesting that membrane-associated proteins were greatly reinforced in DR. The unknown phenomenon in plant stress physiology was defined as a proteome translocation response. Quantitative 2D-DIGE technology highlighted these ‘bound’ proteins in DR. Fifty-eight kinds of proteins were identified as candidates of the translocated proteome in *Phragmites*. The majority were chloroplast proteins. Unexpectedly, Rubisco was the most abundant protein sequestered by DR. Rubisco activase, various chaperons and 2-cysteine peroxiredoxin were major components in the translocation response. Conformational change was assumed to be the main reason for the Rubisco translocation due to no primary sequence difference between DR and SR. The addition of reductant in extraction process partially reversed the translocation response, implying that intracellular redox status plays a role in the translocation response of the proteome. The finding emphasizes the realistic significance of the membrane-association of biomolecule for plant long-term adaptation to complex stress conditions.

## Introduction

Plants are often simultaneously exposed to various stresses due to their sessile lifestyle. For example, plants growing in desert regions tolerate multiple stresses including drought, heat, high light, and nutrient deficiency. Increasing evidence shows that combinations of stresses impact plant development more severely than a single stress (Staiger and Brown, 2013). Plant molecular responses to combined stresses also differs from those to a single stress (Rizhsky et al., 2004; Mittler, 2006; Atkinson and Urwin, 2012). Gene expression under combined stresses cannot be predicted from single stress treatments because some specific bioprocesses/programs might be activated (Rizhsky et al., 2004; Prasch and Sonnewald, 2013; Rasmussen et al., 2013). Therefore, analyzing plant responses to simultaneous multiple stresses, e.g., drought, heat, and high light, using naturally occurring variations is important for elucidating the mechanism of their adaptation to adverse environments.

Physiological and molecular events in plant adaptation to adverse environments have been investigated for decades of years, but most experiments are performed under single stress scenarios, and generally over short-term periods (Garrett et al., 2006). Such stresses imposed in a laboratory commonly result in the deformation of/damage to proteins, DNA, or other essential macromolecules (Kultz, 2003). Meanwhile, diverse molecular mechanisms are triggered, including cell cycle control, redox regulation, protein chaperonin and repair, DNA and chromatin stabilization and repair, modulation of energy metabolism, removal of damaged proteins, and certain aspects of metabolism (Hirayama and Shinozaki, 2010; Krasensky and Jonak, 2012; Singh and Laxmi, 2015). Quantitative transcriptome and proteome analysis can be used to monitor numerous RNAs and proteins involved in these processes. The studies examining a single stress have allowed identification of specific genes linked to the stress factor, but have failed to explain complex plant responses to multifactorial stresses, especially when the stresses were imposed over a long-term basis.

*Phragmites communis* (common reed) is a perennial gramineous wetland species with a cosmopolitan distribution. Typical habitats of the species are lakesides, riversides, and shallow freshwater swamps. However, *Phragmites* has often been observed to tolerate a broad range of adverse environmental conditions such as water-starved terrestrial habitats, including dry riverbeds and reservoirs, and even seriously desertified regions (Gorenflot et al., 1984; Chen and Zhang, 1991). Thus far, there is limited information on the inherent mechanism underlying the broad toleration/adaptation. In northwest China, various ecotypes of *Phragmites* have been described, with special adaptations to distinct site conditions (Chen and Zhang, 1991; Wang et al., 1995). Of these, the ecotypes known as SR (swamp reed) and DR (desert-dune reed) are two sympatric populations, which were derived from long-term evolution in contrasting environments, i.e., aquatic habitats and desert dune habitats, respectively (Zhu et al., 2003). Since they are located within a narrow area (less than 6.5 km^2^), the two sympatric ecotypes of *Phragmites* share similar meteorological conditions (Cui et al., 2009). Therefore, they are an ideal model for studying plant adaptation to long-term multifactorial stresses. Over a span of 20 years, the alteration in morphology and ultrastructure, physiological distinction, variations of cellular components as well as enzyme polymorphisms have been extensively investigated in the two ecotypes of *Phragmites* (Chen and Zhang, 1991; Wang et al., 1995; Zheng et al., 2000; Cheng et al., 2001; Zhu et al., 2001; Chen et al., 2003, 2007; Gong et al., 2003; Zhao et al., 2004; Cui et al., 2005). Compared with SR, DR growing on natural dunes produces short and small shoots, narrow leaves with huge vascular bundles, and a highly lignified and suberized cell wall (Wang et al., 1995; Cui et al., 2009); its efficiency of photosynthesis is dramatically decreased (Zhu and Zhang, 2000), but its antioxidant system is very active (Zhu et al., 2001; Chen et al., 2003; Cui et al., 2009). Recent efforts have been directed at the analysis of differential protein expression between SR and DR. The large-scale proteomics analyses were conducted using quantitative fluorescence difference in gel electrophoresis (DIGE), in combination with MALDI TOF/TOF mass spectrometry. Forty-five differentially expressed proteins in key path ways were identified (Cui et al., 2009). All differentially expressed proteins involved in the light reactions of photosynthesis had a dramatically lower expression, whereas those involved in protein biosynthesis and quality control were usually highly expressed in DR (Cui et al., 2009). In addition, active ascorbate and glutathione metabolism also operated in DR (Cui et al., 2009). Further, while we revealed the proteomic characteristics of the species adaptation to the complex stress environment, we noticed that DR has a peculiarity with respect to protein solubility. The soluble fraction of DR is always maintained at a lower percentage in total proteins, despite its total protein content was similar to SR (Cui et al., 2009). As early as 1992, Ren and Zhang (1992) found that the soluble proteins in DR appeared entirely different protein profile on SDS-PAGE gels when loaded equally with other reed ecotypes. They reported that the high-abundant leaf proteins functioning in photosynthesis were hardly observed in DR, except for a dominant unknown protein with a lower molecular weight. Other investigations confirmed the phenomenon (Wang et al., 1994; Yang et al., 1994). Summarizing these reports and our unpublished data, we speculate that the change in protein solubility is an unknown proteome translocation response, which occurred in the desert-dune ecotypes of *Phragmites* to cope with adverse habitats on a long-term basis.

To illustrate the phenomenon, in this study, we monitored protein changes in the distribution between the soluble and insoluble fractions in the two ecotypes of *Phragmites*.

Data from three sampling years were reported. Using quantitative DIGE technology, an unambiguous map of protein translocation came into view. Based on a comparison with SR, proteins that translocated ≥25% to the insoluble fraction of DR were identified by mass spectrometer. Fifty-eight *Phragmites* proteins significantly altered their solubility in DR. Ribulose-1,5-bisphosphate carboxylase/oxygenase (Rubisco), a key enzyme in the dark reaction of photosynthesis, was one of the major components in the translocation proteome. Given its considerable abundance and translocation rate, we sequenced its primary cDNA and analyzed its amount and activity in the absence and presence of a reducing agent to determine the reasons for the proteome translocation. We deduced that the proteome translocation response that occurred in the desert plant was partially reversible and regulated by intracellular redox status. The large-scale protein translocation could be a protective strategy for the long-term adaptation of the plant to complex stress environments.

## Materials and Methods

### Plant Materials

Two ecotypes of *Phragmites communis*, i.e., swamp reed and desert-dune reed, were obtained in southern margin of the Badan jilin desert in Northwest China. Swamp reed (SR) naturally grows in a nameless rivulet; desert-dune reed (DR) grows on natural sand dunes (Wang et al., 1995; Cui et al., 2009). They are typical sympatric populations distributing a narrow area with about 6.5 km^2^ (39°31′–58′N, 100°4′–36′E; elevation 1300 m). The mean annual precipitation of the sampling site is 118 mm, with the annual potential evaporation of 2392 mm and daily fluctuation of air temperature (data from local Meteorological Bureau). Every year, the leaf tissues of the two ecotypes were collected at the beginning of June. For avoiding single clone, nine plots with 50 m in distance were selected for three biological replicates of each ecotype (Supplementary Figure S1). After harvesting, the samples were rapidly frozen and stored at -80°C for protein extraction, or immediately used for physiological analysis.

### Water Status

To evaluate the water status in leaf tissues, three physiological indicators, i.e., water content, levels of free and bound water, water potential, were determined in the field. The water content was calculated as the difference between the fresh and dry masses and expressed on a fresh weight basis. The levels of both free and bound water were determined according to the method described (Zhou, 2000). Water potentials were directly monitored using a dew-point water potential meter (WP4C, Decagon, USA). For every assay, three replicates were performed.

### Fractional Extraction of Both Soluble and Insoluble Proteins

Protein fractionation was performed according to a previous protocol (Cui et al., 2005, 2009) with modifications. Leaf tissues (1.0 g) from SR and DR were ground, respectively, to a fine powder in liquid nitrogen. For the soluble fraction of the proteins, extraction buffer (50 mM Tris-HCl, pH 7.8, 10% glycerol, 2% β-mercaptoethanol, 1 mM PMSF, 1 mM EDTA) was added and homogenized. Ultrasonic crushing was carried out under the condition of 10 cycles of 10 s/10 s to promote protein extraction. After centrifugation at 43,600 × *g* for 30 min, the supernatant containing the soluble proteins was collected into a new test tube. The pellet was frozen again in liquid nitrogen and ground in the same buffer. After ultrasonication and centrifugation, the resulting supernatant was pooled into the test tube. Following the removal of the soluble proteins, the remaining pellet was extracted for insoluble proteins. Specifically, the pellet was suspended in 100 mM phosphate buffer (pH 7.1) containing the detergent CHAPS (4% w/v), 0.2 M KCl, 2 mM MgSO_4_, 10% glycerol, 2% β-mercaptoethanol, 1 mM PMSF and 1 mM EDTA. After ultrasonication and incubation at 4°C for 30 min, 7 M urea, 2 M thiourea and 30 mM DTT were added and incubated for an additional 30 min at room temperature. After centrifugation at 18°C, the supernatant was collected as the first part of the insoluble fraction. Further, the remaining pellet was re-suspended in a solution containing ASB-14, TBP, and ampholyte (3/10) for obtaining more insoluble proteins. After centrifugation, the second supernatant was pooled together with the above protein solution as an intact insoluble fraction. For purifying protein samples, phenol extraction coupled with saturated ammonium acetate precipitation was applied as described previously (Cui et al., 2012). The protein concentration in the fractions was determined using our established method (Cui et al., 2009). Unless otherwise stated, all extraction steps were carried out at 4°C.

### Protein Cydye Labeling

For labeling with DIGE dyes, lyophilized protein samples were re-suspended in lysis buffer, and then labeled reciprocally with Cy3 and Cy5 according to the manufacturer’s instructions (GE Healthcare). An internal standard generated by pooling equal amounts of proteins from each sample were labeled with DIGE-specific Cy2 (Supplementary Table S1). Three biological replicates (including 12 samples) were carried out in the experimental set.

### 2-D DIGE and Image Analysis

Pairs of Cy3- and Cy5-labeled protein samples and a Cy2-labeled internal standard were mixed together and subjected to IEF (24-cm IPG strips at pH 4-7, GE Healthcare) and SDS-PAGE, as described previously (Cui et al., 2009, 2012). After scanning using the Typhoon 9400 Imager (GE Healthcare), the DIGE images were analyzed using DeCyder v6.5 software. Spot intensities were normalized based on the internal standard labeled by Cy2. The ratios of soluble and insoluble proteins were obtained for each reed ecotype using the DeCyder software. Based on the ratios (Student’s *t*-test, *P* < 0.05), the proportion of each protein spot in the soluble and insoluble fraction was calculated. The rate of protein translocation, i.e., the difference of insoluble fraction between SR and DR, was obtained. The protein spots with more than 25% in translocation rate were selected and subjected to MS identification.

### In-gel Tryptic Digestion, Mass Spectrometry, and Database Search

For protein identification, traditional 2-DE gels stained with Coomassie brilliant blue (CBB) were prepared using the same parameters (see Supporting Information). The spots of interest were picked and submitted to in-gel digestion with trypsin. After washing and dehydrating, the spots were covered with trypsin solution (12.5 ng/μl in 50 mM NH_4_HCO_3_) for 45 min in ice. Trypsin digestion was performed overnight at 37°C and stopped by adding 5% formic acid. Extracted peptides were analyzed by MALDI TOF/TOF MS (Ultraflex III, Bruker Daltonics, Germany) as previously described (Cui et al., 2012). In brief, samples were spotted onto a MALDI target plate using cyano-4-hydroxycinnamic acid matrix. Mass data acquisitions were piloted automatically by an auto Xecute method within the Flex Control software v3.0. In MS mode, spectra were acquired in a mass range m/z 800–4500 by summing up 2000 laser shots with an acceleration of 23 kV. The MS spectra were externally calibrated using Peptide Calib Standard II (Bruker Daltonics) (1046.542, 1296.685, 1347.735, 1619.822, 2093.086, 2465.198, and 3147.471), resulting in mass errors of <50 ppm. The MS peaks were detected with a minimum signal/noise (S/N) ratio > 20 and cluster area S/N threshold > 25 without smoothening and raw spectrum filtering. Peptide precursor ions corresponding to contaminants including keratin and the trypsin autolytic products were excluded in a mass tolerance of 0.2 Da. For acquiring MS/MS spectra, the filtered precursor ions with a user-defined threshold (S/N ratio > 50) were selected. Fragmentation of precursor ions was performed using the LIFT positive mode. MS/MS spectra were accumulated from 4000 laser shots. The MS/MS peaks were detected on a minimum S/N ratio > 3 and a cluster area S/N threshold > 15 with smoothing. Mass spectra were piloted using Flex Analysis software.

The obtained MS and MS/MS spectra per spot were combined, and submitted to MASCOT search engine by Biotools 3.1 (Bruker Daltonics). Parameters selected included: MS/MS Ion Search, the NCBInr database of green plants created on 31 July 2016 (91579380 sequences; 33741759339 residues), trypsin of the digestion enzyme, up to one missed cleavage site, parent ion mass tolerance at 100 ppm, MS/MS mass tolerance of 0.5 Da, carbamidomethylation of cysteine (global modification), and methionine oxidation (variable modification). The probability score (95% confidence level) calculated by the software, and a matching of at least 2 peptides was used as a criterion for correct identification. The MS/MS data set for all proteins are provided in Supplementary Table S2.

### Gene Cloning and Sequencing for Large and Small Subunits of Rubisco

To obtain the full-length cDNA of the *rbcL* and *rbcS* genes, DOP-PCR (degenerated oligonucleotideo-primed-PCR), 3′-RACE (rapid-amplification of cDNA ends) and 5′ TAIL-PCR (Thermal asymmetric interlaced polymerase chain reaction) were performed by using the double-stranded cDNA of SR and DR as a template, respectively. All primers, conditions and cycle settings for PCR were provided in Supplementary Tables S3, S4. The resulting PCR products were ligated into vector TA, cloned, and sequenced. Every sequence was confirmed by examining both strands; in addition, at least two bacterial clones obtained by TA cloning were examined. By assembling the sequences of 3′, 5′ and the core partial sequences on Contig Express (Vector NTI Advance11.5.1), the full-length cDNA sequence of *rbcL* and *rbcS* gene was deduced. The nucleotide sequence data for *rbcL* and *rbcS*, from SR and DR, have been deposited in the GenBank nucleotide sequence databases under accession no. KF697233, KF697234, KF697235, and KF697236, respectively. Finally, the sequence alignments between SR and DR were determined using AlignX (Vector NTI Advance11.5.1) (see Supplementary Figure S2).

### Rubisco Quantification and Activity Assay

For determining both the amount and activity of Rubisco, leaf soluble proteins were obtained from SR and DR using extraction buffer containing 40 mM Tris-HCl (pH 7.6), 10 mM MgCl_2_, 0.25 mM EDTA, 5 mM glutathione, 2% (v/v) β-mercaptoethanol and a small amount of PVP-40. The extraction process was repeated once, and the resulting supernatants were pooled. In the process, no supersonic step was conducted to avoid loss of the enzyme activity. For quantifying the Rubisco content specifically, proteins from the supernatant were precipitated with methanol/chloroform according to the method of Wessel and Flügge (1984). IEF and SDS-PAGE for 2-DE were carried out as described previously (Cui et al., 2009). Specifically, only 200-μg protein samples were loaded onto 24-cm IPG strips (pH 4-7) in order to alleviate the saturation effect of the highly abundant protein. The resulting 2D gels were stained using CBB and digitized with a calibrated densitometer (GS-800, Bio-Rad). The images were analyzed using PDQuest software v7.1.1 (Bio-Rad). The intensity level of the Rubisco protein (including the large and small subunits) was obtained by expressing the intensity of each spot in a gel as a proportion of the total protein intensity detected using the entire gel. The Rubisco contents were calculated based on the loading amount.

Rubisco activity was measured by rate of NADH oxidation at 340 nm according to a method described (Reid et al., 1997) with minor modifications. Briefly, the initial Rubisco activity was measured at 340 nm by adding 50 μL of the supernatant to 950 μL of assay buffer containing 40 mM Tris-HCl (pH 7.6), 0.25 mM EDTA-Na_2_, 10 mM MgCl_2_, 5 mM glutathione, 0.2 mM NaHCO_3_, 5 mM ATP, 0.5 mM NADH, 5 mM creatine phosphate, 0.5 mM RuBP, 10 units of phosphocreatine kinase, 10 units of glyceraldehydes-3-phosphate dehydrogenase and 10 units of phosphoglycerate kinase. All regents listed above were obtained from Sigma (USA). Subsequently, the mixture was measured at 340 nm every 30 s for 3 min. Rubisco activity was expressed in CO_2_ nmol min^-1^ mg^-1^Fresh weight/Soluble proteins/Rubisco.

### Effect of the Reducing Agent on Rubisco Content and Activity

According to the method above, soluble proteins were extracted in the presence or absence of the reducing agent β-mercaptoethanol (ME) buffer. The amount and activity of Rubisco were determined. Three biological repeats were carried out for evaluating the effect of the reductant on the different distribution of Rubisco in SR and DR.

## Results

### Morphometric Characteristics

**FIGURE 1:**
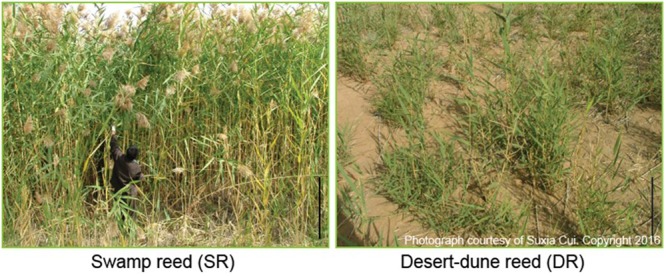
**Growth and morphological characteristics of two ecotypes of *Phragmites communis* in their habitats.** Swamp reeds (SR) grow in a rivulet; Desert-dune reeds (DR) inhabit natural desert dunes. They are sympatric population located less than 5 km apart. Bar: 1 m. The photographs were taken by Suxia Cui in 2009.

The two ecotypes of *Phragmites*, SR and DR, are typical sympatric populations with contrasting habitats of water availability (Cui et al., 2002, 2009). **Table 1** lists five morphometric parameters measured at the mature plant stage to evaluate the effect of environmental stresses on growth and development. Significant differences between SR and DR were evident for all five traits (*P* < 0.01). Undoubtedly, harsh desert habitats lead to growth retardation of DR, resulting in a 3.2 and 10.6-fold reduction in height and biomass, respectively, in comparison to SR with a good water supply (**Figure 1** and **Table 1**).

**Table 1:** Growth index of *Phragmites* ecotypes at the mature stage (Data were collected on September, 2012).

Ecotypes	Shoot height (m)	Leaf number	Flag-leaf length (cm)	Flag-leaf width (cm)	Biomass (Kg Fw/m^2^)	Biomass per plant (g Fw/plant)
Swamp reed	3.93 ± 0.68^aA^	17 ± 4^a^	41.4 ± 2.1^aA^	2.7 ± 0.1^aA^	5.73 ± 1.51^aA^	181 ± 44^aA^
Desert-dune reed	1.24 ± 0.21^bB^	13 ± 1^b^	18.0 ± 4.6^bB^	1.0 ± 0.1^bB^	0.30 ± 0.06^bB^	17 ± 4^bB^

*Results are expressed as the mean ± SE (*n* = 3 separate determinations). A and B indicates *p* < 0.01; a and b indicates *p* < 0.05. Statistical analysis was performed using Student’s two-tailed *t*-test.*

### Leaf Water Status

**FIGURE 2:**
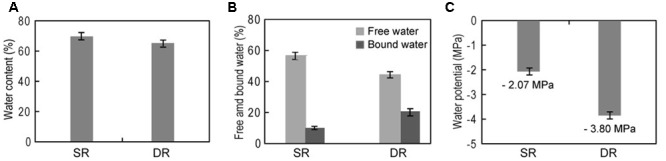
**Leaf tissue water status of two *Phragmites* ecotypes. (A)** Leaf water content; **(B)** both free and bound water; **(C)** water potential. SR, swamp reed; DR, desert-dune reed.

Considering the huge difference in the water supply of the habitats, the leaf water content of both SR and DR was carefully measured. Unexpectedly, the water contents were highly similar, i.e., 61.2% (SR) and 60.8% (DR) (**Figure 2A**). There were no significant differences in any of the leaves collected from any leaf position. The similarity did not match the moisture state in their habitats. To explore the inherent reason for this result, we aimed to test the water activity through two independent methods. First, water components (free water and bound water) were measured (**Figure 2B**). As expected, the free water was remarkably decreased in DR, with a concomitant increase in the bound water. The level of bound water in DR (31.9%) was more than twofold higher than that in SR (15.1%). Second, we monitored the leaf water potential in a real time in the field using a portable water potential meter (WP4C). Compared with the potential of -2.07 MPa in SR, the DR value was more negative (-3.8 MPa) (**Figure 2C**), implying that the water molecules were bound to other biomolecules in DR. Therefore, we conclude that the water activity in DR was down-regulated for coping with the water deficit in its habitat. The underlying causes of the strategy need to be investigated.

### Protein Fractionation Assay

In our previous proteomic research, a step-by step workflow extraction was established for obtaining entire proteins from the leaves of *Phragmites* (Cui et al., 2009). We noticed that the protein contents were similar between SR and DR, but the percentages of the soluble and insoluble proteins were dramatically altered in the two ecotypes. Compared with SR, DR exhibited a very low level of soluble proteins, and a high level of insoluble proteins (Cui et al., 2009). The alteration in the protein components showed a similar trend as that observed for the water components.

**FIGURE 3:**
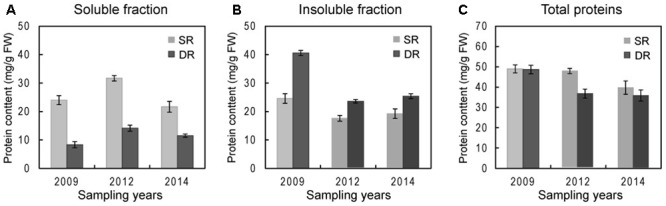
**Protein contents in both the soluble and insoluble fractions in two ecotypes of *Phragmites*. (A)** Soluble protein contents, **(B)** insoluble protein contents, and **(C)** total protein contents in annual sampling was carried out in early June. Leaf proteins from SR and DR were separated using the step-by step extraction strategy described in section “Materials and Methods.”

To confirm the finding and decipher the underlying meaning of the information, we collected reed leaf samples from their natural habitats in 2009, 2012, and 2014 for the current protein fractionation assay. The fractional protocol was optimized for maintaining the integrity of each protein fraction (see Materials and Methods; Supplementary Figure S3). **Figure 3** shows the results that were generated from three sampling years in 2009, 2012, and 2014. Based on the optimized fractionation method and dozens of experiments, we confirmed that the soluble proteins in DR accounted for about 28.7% (on average) of total proteins, a lower level compared with 55.6% for SR. On the other hand, the insoluble proteins in DR appeared to exhibit an increasing trend, i.e., 71.4% on average (**Figure 3**). We assume that regulation of the protein proportion between the soluble and insoluble fractions plays a role in DR resistance to environmental stresses.

### Protein Profiles of both Soluble and Insoluble Fractions

**FIGURE 4:**
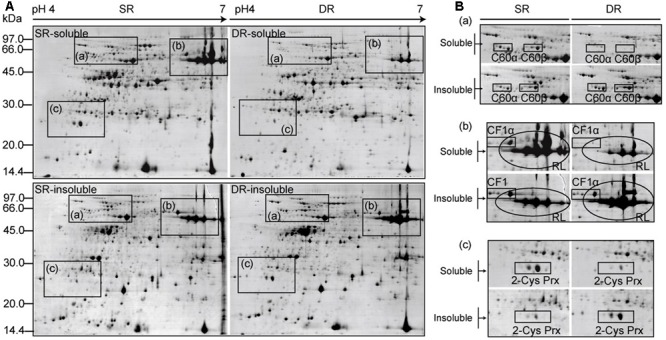
**Protein profiles of both soluble and insoluble fractions in two ecotypes of *Phragmites*. (A)** Proteins were separated using IPG strips (pH4-7, 24 cm) and a 12.5% SDS-PAGE gel. The gels were stained with CBB R-250. **(B)** The enlarged diagrams correspond to boxes from **(A)**. C60α and C60β: alpha and beta subunits of the chaperonin 60 kDa protein; RL, rubisco large subunit; CF1α, ATP synthase CF1 alpha chain; 2-Cys Prx, 2-Cystenin peroxiredoxin.

For analyzing the protein proportion alteration, high-resolution 2D gels were generated within a pH range of 4–7 based on 24-cm IPG strips. **Figure 4A** shows the four protein profiles corresponding to each fraction of DR and SR. We observed that numerous, highly abundant proteins, which were identified in our previous paper (Cui et al., 2009), were shared in two fractions, but with varying proportions (**Figure 4A**). For instance, two subunits (C60α and C60β) of the chloroplast chaperonin 60 kDa were mainly distributed in the soluble fraction of SR, but emerged dominantly in the insoluble fraction of DR (**Figure 4Ba**); the large subunit of Rubisco (RL) and the alpha subunit of ATP synthase (CF1α) were located mainly in the soluble fraction of SR, but were highlighted in the insoluble fraction of DR (**Figure 4Bb**), as was 2-cystenin peroxiredoxin (2-Cys Prx) (**Figure 4Bc**).

**FIGURE 5:**
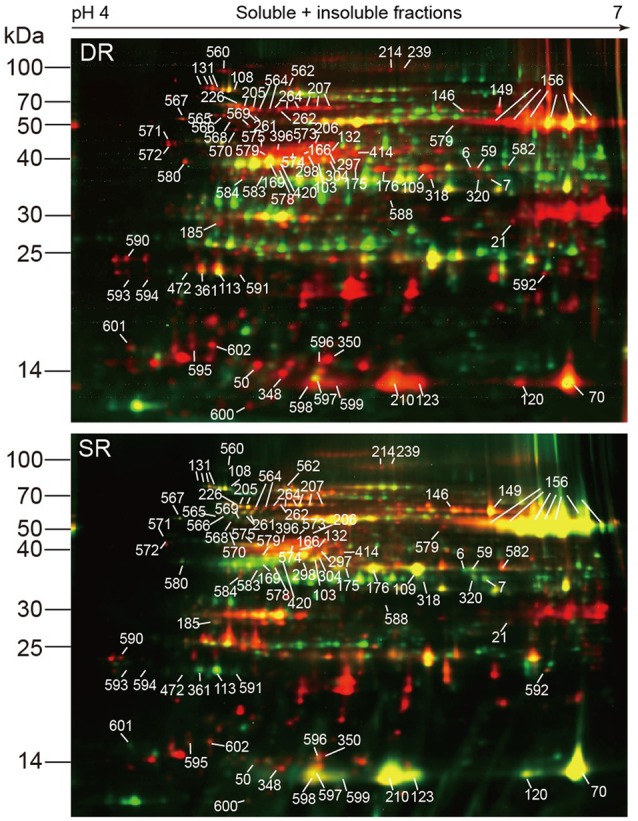
**Representative DIGE maps show the difference between the protein fractions in two ecotypes of *Phragmites*.** The DIGE gels were generated within a pH range of 4–7 using 24-cm IPG strips. The soluble and insoluble proteins from SR and DR were labeled using Cy3 (green) and Cy5 (red), respectively. Mixed proteins used as an internal standard was labeled using Cy2. Marked protein spots were those shifted more than 25% to the insoluble fraction of DR in comparison to SR.

For obtaining accurate quantitative information on the alteration in protein distribution, we further designed and performed a comprehensive proteomic analysis using the DIGE technique. Pairs of Cy3- and Cy5-labeled proteins from the soluble and insoluble fractions, as well as a Cy2-labeled internal standard were mixed together and then subjected to IEF and SDS-PAGE. Triplicates with independent biological samples were carried out in the experimental set. Two representatives of the DIGE maps, corresponding to SR and DR, are shown in **Figure 5**. Using the DeCyder v6.5 BVA (Biological Variation Analysis) module, approximately 1730 protein spots were detected on the DIGE gels. Based on the soluble/insoluble protein ratios in each reed ecotype, the protein translocation rate, i.e., the value of the translocating into the insoluble fraction of DR in comparison to SR, was calculated and listed in **Table 2** and Supplementary Table S5. A user-defined threshold (translocation ≥ 25%) was employed to identify the translocation proteins.

**Table 2:** Distribution percentages of proteins between the soluble and insoluble fractions.

No.	Protein name	Spot no.	Fractions in SR			Fractions in DR			Translocation	Predicted
			Sol.%	Inso. %	*p*-value^a^	Sol. %	Inso. %	*p*-value^a^	DR_inso_- SR_inso_ (%)	localization
**Photosynthesis**										
(1)	Rubisco large subunit	156-1^b^	56.4	43.6	0.015	12.2	87.8	0.031	*44.1*	Chloroplastic
		156-2	79.8	20.2	0.009	7.2	92.8	0.048	*72.6*	
		156-3	57.7	42.3	0.020	13.5	86.5	0.045	*44.2*	
		156-4	60.6	39.4	0.045	11.7	88.3	0.039	*48.9*	
		156-5	70.9	29.1	0.022	18.4	81.6	0.017	*52.5*	
		156-6	75.8	24.2	0.039	21.6	78.4	0.045	*54.2*	
		*Average*	*66.9*	*33.1*		*14.1*	*85.9*		*Average 52.8*	
		21	46.7	53.3	0.015	10.0	90.0	0.018	*36.8*	
		50	42.4	57.6	0.028	10.5	89.5	0.020	*31.9*	
		348	27.7	72.3	0.008	3.7	96.3	0.001	*24.0*	
		350	28.6	71.4	0.015	4.3	95.7	0.001	*24.3*	
(2)	Rubisco small subunit	210	64.9	35.1	0.024	15.4	84.6	0.019	*49.6*	Chloroplastic
		123	71.8	28.2	0.047	15.0	85.0	0.023	*56.7*	
		120	62.5	37.5	0.038	11.3	88.7	0.004	*51.2*	
		70	63.4	36.6	0.028	11.8	88.2	0.034	*51.7*	
		*Average*	*65.7*	*34.3*		*13.4*	*86.6*		*Average 52.3*	
(3)	Rubisco activase	414	66.3	33.7	0.044	11.0	89.0	0.019	*55.4*	Chloroplastic
		297	33.7	66.3	0.030	3.5	96.5	0.042	*30.2*	
		298	70.4	29.6	0.006	37.9	62.1	0.021	*32.5*	
		396	49.0	51.0	0.002	3.5	96.5	0.015	*45.5*	
(4)	Rubisco activase	103	58.3	41.7	0.015	16.8	83.2	0.010	*41.5*	Chloroplastic
		304	50.0	50.0	0.004	10.9	89.1	0.079	*39.1*	
(5)	Rubisco activase	109	65.9	34.1	0.021	5.7	94.3	0.016	*60.2*	Chloroplastic
		176	61.5	38.5	0.017	9.5	90.5	0.019	*52.1*	
		175	65.5	34.5	0.002	21.3	78.7	0.005	*44.2*	
(6)	Rubisco activase	320	67.8	32.2	0.007	20.3	79.7	0.007	*47.5*	Chloroplastic
		*Average*	*58.8*	*41.2*		*14.0*	*86.0*		*44.8%*	
(7)	Glyceraldehyde-3-phosphate dehydrogenase	318	62.4	37.6	0.028	11.5	88.5	0.016	*50.9*	Chloroplastic
(8)	Fructose-1,6-bisphosphatase	6	67.6	32.4	0.019	20.9	79.1	0.012	*46.7*	-
(9)	Phosphoribulokinase	169	81.5	18.5	0.011	53.5	46.5	0.015	*28.0*	Chloroplastic
		420	75.7	24.3	0.015	51.2	48.8	0.010	*24.5*	
**Transport ATPases**										
(10)	ATP synthase α chain	149	41.8	58.2	0.029	3.7	96.3	0.041	*38.1*	Chloroplastic
		146	43.1	56.9	0.018	11.9	88.1	0.004	*31.2*	
(11)	ATP synthase β chain	166	37.9	62.1	0.015	5.4	94.6	0.015	*32.5*	Chloroplastic
(12)	ATP synthase 𝜀 subunit	596	46.1	53.9	0.015	17.9	82.1	0.001	*28.2*	Chloroplastic
**Protein synthesis**										
(13)	40S ribosomal protein S16	567	63.0	37.0	0.022	26.6	73.4	0.002	*36.4*	Chloroplastic
(14)	50S ribosomal protein L10	592	49.8	50.2	0.032	14.0	86.0	0.006	*35.8*	Chloroplastic
(15)	50S ribosomal protein L31	600	39.2	60.8	0.007	7.4	92.6	0.007	*31.8*	Chloroplastic
(16)	Chloroplastic 30S ribosomal protein S1-like	571	30.3	69.7	0.007	3.8	96.2	0.001	*26.5*	Chloroplastic
(17)	Translational elongation factor Tu	206	35.8	64.2	0.005	9.7	90.3	0.010	*26.1*	Chloroplastic
		573	40.0	60.0	0.018	14.5	85.5	0.004	*25.5*	
**Protein folding and stability**										
(18)	Chaperonin 60 α subunit	205-1^b^	61.1	38.9	0.043	16.7	83.3	0.001	*44.4*	Chloroplastic
		205-2	63.9	36.1	0.039	11.9	88.1	0.001	*52.0*	
(19)	Chaperonin 60 α subunit	226	63.4	36.6	0.048	19.7	80.3	0.058	*43.7*	Chloroplastic
(20)	Chaperonin 60 α subunit	262	53.3	46.7	0.029	10.6	89.4	0.013	*42.7*	Chloroplastic
		*Average*	*60.4*	*39.6*		*14.7*	*85.3*		*Average 45.7*	
(21)	Chaperonin 60 β subunit	207-1^b^	62.1	37.9	0.014	4.7	95.3	0.031	*57.5*	Chloroplastic
		207-2	58.7	41.3	0.007	14.0	86.0	0.024	*44.7*	
		207-3	49.5	50.5	0.019	17.3	82.7	0.014	*32.2*	
(22)	Chaperonin 60 β subunit	264-1^b^	47.6	52.4	0.043	5.6	94.4	0.007	*42.0*	Chloroplastic
		264-2	56.3	43.7	0.031	2.7	97.3	0.009	*53.6*	
		264-3	58.0	42.0	0.028	3.3	96.7	0.016	*54.7*	
(23)	Chaperonin 60 β subunit	261-1^b^	66.9	33.1	0.042	22.4	77.6	0.013	*44.5*	Chloroplastic
		261-2	64.2	35.8	0.008	18.7	81.3	0.038	*45.4*	
(24)	Chaperonin 60 β subunit	564-1^b^	54.1	45.9	0.017	14.8	85.2	0.013	*39.4*	Chloroplastic
		564-2	53.1	46.9	0.034	10.2	89.8	0.037	*42.9*	
		*Average*	*57.1*	*43.0*		*11.4*	*88.6*		*Average 45.7*	
(25)	70 kDa heat shock-like protein	131-1^b^	55.4	44.6	0.003	21.8	78.2	0.004	*33.6*	Chloroplastic
		131-2	57.3	42.7	0.003	16.8	83.2	0.005	*40.5*	
		131-3	57.8	42.2	0.007	21.0	79.0	0.003	*36.8*	
		131-4	64.5	35.5	0.032	30.7	69.3	0.036	*33.9*	
		*Average*	58.8	41.3		22.6	77.4		*Average 36.2*	
(26)	70 kDa HSP	108	67.2	32.8	0.003	36.8	63.2	0.006	*30.4*	Chloroplastic
(27)	90 kDa heat shock-like protein	560	59.7	40.3	0.029	11.6	88.4	0.0051	*48.0*	
(28)	Peptidyl-prolyl cis-trans isomerase	580	67.0	33.0	0.041	29.3	70.7	0.003	*37.7*	Chloroplastic
(29)	lumenal binding protein	562	62.5	37.5	0.017	31.4	68.6	0.016	*31.1*	Secretory
(30)	ATP-binding subunit of ATP- dependent Clp protease	214	41.3	58.7	0.034	15.2	84.8	0.035	*26.1*	Chloroplastic
		239	49.5	50.5	0.006	24.4	75.6	0.039	*25.1*	
**Disease/defense**										
(31)	2-Cystenin peroxiredoxin	472	65.2	34.8	0.017	27.3	72.7	0.001	*37.8*	Chloroplastic
		361	72.6	27.4	0.025	32.3	67.7	0.019	*40.3*	
		113	66.2	33.8	0.007	31.2	68.8	0.001	*35.1*	
		591	64.3	35.7	0.016	35.3	64.7	0.022	*29.0*	
		*Average*	67.1	32.9		31.5	68.5		*Average 35.6*	
(32)	Peroxiredoxin-2E-2	602	35.1	64.9	0.006	9.7	90.3	0.001	*25.4*	Chloroplastic
(33)	Thioredoxin M	597	56.1	43.9	0.049	24.3	75.7	0.000	*31.8*	Chloroplastic
(34)	Plastidic glutamine synthetase	132	34.7	65.3	0.015	5.1	94.9	0.004	*29.7*	Chloroplastic
		574	50.2	49.8	0.003	15.8	84.2	0.007	*34.5*	
**Others**										
(35)	14-3-3-like protein	185	55.0	45.0	0.034	25.6	74.4	0.027	*29.3*	-
(36)	Adenosine kinase-like protein	579	71.8	28.2	0.006	44.8	55.2	0.001	*26.9*	-
(37)	mRNA binding protein	7	69.2	30.8	0.011	41.8	58.2	0.006	*27.4*	Chloroplastic
(38)	RNA-binding protein	598	60.8	39.2	0.023	15.9	84.1	0.007	*44.9*	-

*Italicized font means core data in this experiment.*

*^a^*P*-value (<0.05) shows *T*-test of sol/inso ratio for each protein spot, from built-in analysis of DeCyder software v6.5.*

*^b^For clarity, some proteins with multiple isoforms were labelled using numerals, corresponding to spots (from left to right) on maps (**Figure 5**).*

*Those translocated ≥ 25% into the insoluble fraction of DR and their functional categories are listed. A total of 74 protein spots were identified, corresponding to 39 proteins by combining PMF information and at least two peptide sequences using MALDI-TOF/TOF MS according to a previous method (Cui et al., 2009). The localization predictions were carried out using TargetP (http://www.cbs.dtu.dk/services/TargetP/).*

### Protein Identification and Functional Classification

In comparison to SR, 93 protein spots shifted ≥ 25% into the insoluble fraction of DR (**Figure 5**). The majority of the protein spots (about 80%) were identified by combined analysis of MALDI TOF/TOF MS and databases using the previous method (Cui et al., 2009). Supplementary Table S2 describes the detailed information pertaining to the protein identification. Based on the established features of metabolism (Buchanan et al., 2015) and the functional classification of the Gene Ontology^1^, the proteins were classified into five functional categories, i.e., photosynthesis, transport ATPases, protein synthesis, protein folding and stability, as well as disease/defense (**Table 2**). Nineteen proteins with an unknown function could not be identified using current databases (see Supplementary Table S5).

**FIGURE 6:**
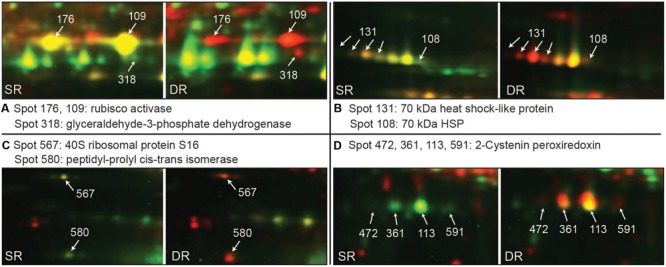
**Examples of proteins translocation to the insoluble fraction in *Phragmites* ecotypes (data from **Figure 5**).** Pairs of Cy3-labeled soluble proteins (green) and Cy5-labeled insoluble proteins (red) were mixed together and subjected to 2D-DIGE. Proteins with high soluble status appear green, while those with high insoluble status appear red. Using MALDI TOF/TOF MS, all marked proteins were identified. **(A–D)** Shows most obvious translocated spots.

Six proteins are involved in the dark reaction of photosynthesis (**Table 2**). The most conspicuous change occurred in Rubisco, the chloroplast protein with the greatest relative abundance (six isoforms in spot 156 and spots 210, 123, 120, 70). We found that the majority (66.9 and 65.7%) of the large and small subunits of Rubisco was distributed in the soluble fraction of SR; the remainder (33.1 and 34.3%) was in the insoluble fraction of SR. The two-phase distribution could be associated with the stroma and thylakoid membrane of the chloroplast (Jin et al., 2006). That is, most of the Rubisco in SR was located and functioned in the stroma of the chloroplast, whereas only a small amount was located in the thylakoid membrane. On the contrary, only 14.1 and 13.4% of the large and small subunits of Rubisco were distributed in the soluble fraction of DR compared with 85.9 and 86.6%, respectively, in the insoluble fraction of DR. By contrast, 52.8 and 52.3% of large and small subunits of Rubisco shifted, respectively, to the insoluble fraction of DR. The distribution result indicates that the majority of Rubisco was sequestered in the thylakoid membrane of DR. Interestingly, the protein translocation mainly occurred in the main product with an observed molecular weight of 54 KDa (spots 156). Another protein Rubisco activase (RCA), an activator of Rubisco, had similar behavior (**Figures 5**, **6A**). At least 4 isoforms of RCA (10 spots) with different molecular weights and isoelectronic points altered their binding status in the chloroplasts (**Table 2**). Soluble RCA in SR accounted for about 58.8% (on average), whereas the proportion decreased significantly to 14.0% in DR. The result also implied that the majority of RCA was attached to the thylakoid membrane of DR. As early as 2001, a similar translocation response was reported in spinach leaves during heat treatment (Rokka et al., 2001). In addition, a proportional change in RCA in the stroma and thylakoids has been observed in antisense *rca* rice (Jin et al., 2006). We speculate that the distribution of RCA in the chloroplasts is dynamically regulated based on alterations in environmental conditions. Other enzymes functioning in the dark reaction of photosynthesis, including glyceraldehyde-3-phosphate dehydrogenase (spot 318, **Figure 6A**), fructose-1,6-bisphosphatase (spot 5), and phosphoribulokinase (spots 169, 420), exhibited similar dynamic changes in DR (**Table 2**). In addition, three subunits of ATP synthase, i.e., α (spots 149, 146), β (spot 166), and 𝜀 subunit (spot 596), also exhibited the dynamic changes. Because the three subunits are components of the soluble catalytic core F_1_, we suspect that the change in the fractions reflects a model of activity regulation of the ATPase complex.

**FIGURE 7:**
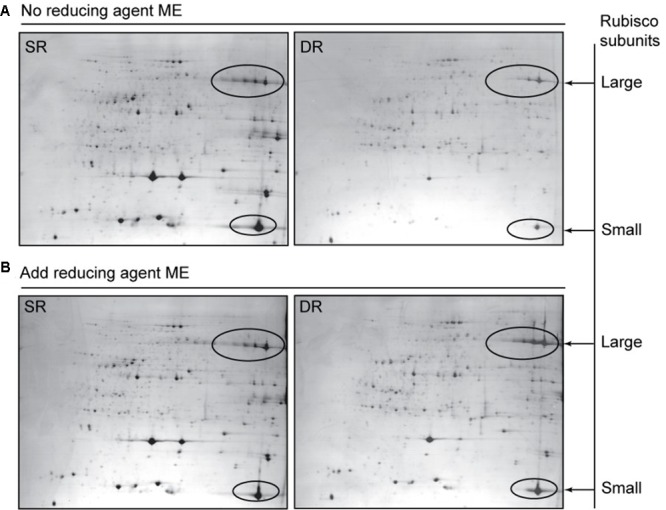
**Soluble protein profiles show the effect of the reductant ME on protein extraction in *Phragmites* ecotypes.** The soluble proteins were extracted in the absence **(A)** or presence **(B)** of ME. In order to alleviate the saturation effect of highly abundant leaf proteins, only 200-μg protein samples were separated based on the IPG strips (24 cm, pH 4-7) and 12.5% SDS-PAGE gel. The gels were stained with CBB R-250. Rubisco contents were calculated according to the intensity levels of the Rubisco protein spots, including the large and small subunits, which are circled on the maps.

Twelve proteins identified are involved in protein synthesis, folding, and stability. In the category of protein synthesis, four proteins (spots 567, 592, 600, and 571) belong to ribosomal protein, and one protein is the translational elongation factor Tu (spots 206, 573). Compared with SR, these proteins apparently translocated (>25%) into the insoluble fraction of DR (**Table 2**). Notably, eight proteins functioning in protein folding and stability also exhibited altered distribution proportions in the soluble and insoluble fractions (**Table 2**). They are α and β subunit 60-kDa chaperonin (Cpn60, 14 spots), two HSPs with 70 kDa (spots 131, 108, **Figure 6B**) and a 90-kDa HSP (spot 560), as well as peptidyl-prolyl *cis–trans* isomerase (PPI, spot 580, **Figure 6C**), lumenal binding protein (spot 436) and the ATP-binding subunit of ATP-dependent Clp protease (spots 214, 239). With respect to Cpn60, we identified at least 3 isoforms of Cpn60-α and 4 isoforms of Cpn60-β according to differences in molecular weight (**Table 2**). Compared with SR, the two subunits of Cpn60 in DR exhibited a significantly altered proportion between the soluble and insoluble fractions. About 45.7% of the proteins were transferred to the insoluble fraction in DR. Although there is evidence that Hsp60 in animal cells undergoes changes in subcellular compartments under stress conditions (Itoh et al., 2002), the inherent physiological function is unknown in plant cells.

In the categories of disease/defense, four chloroplast proteins were found to translocate evidently into the insoluble fraction in DR. They are 2-Cystenin peroxiredoxin (2-Cys Prx, four spots), peroxiredoxin-2E-2 (spot 602), thioredoxin M (spot 597) and plastidic glutamine synthetase (GS2, spots 132, 574). Plant 2-Cys Prx is a key participant in ROS responses, reduce alkyl hydroperoxidase, hydrogen peroxide and even complex lipid peroxides (König et al., 2003). The chloroplast protein usually presented in the form of 3–4 isoforms on a 2D map (**Figures 4Bc**, **6D**), revealed a 35:65% distribution between the soluble and insoluble fractions in SR, and a 10:90% distribution in DR. Compared with SR, the protein translocated about 35% (mean of four isoforms) to the insoluble fraction of DR (**Table 2**). Two other proteins, peroxiredoxin-2E-2 and thioredoxin M, are also two components linked to ROS responses. They are also dominantly distributed in the insoluble fraction of DR. Considering the oxidizing environment caused by environmental stresses in DR cells, the hydrophobization of proteins associated with ROS metabolism might be positive for protection itself.

In addition, a 14-3-3 protein (spot 185) and an adenosine kinase-like protein (spot 579) as well as two RNA binding proteins (spots 7 and 598) presented a significant translocation into the insoluble fraction of DR. Nineteen protein with an unknown function could not be identified using current databased (Supplementary Table S5). Their roles in the translocation sub-proteome need to be characterized in future.

### Role of the Reductant in the Response of Protein Translocation

To provide an explanation for the protein translocation, Rubisco was selected for the following experiments because of its abundance and high translocation rate (**Figure 5** and **Table 2**). By gene cloning and sequencing, full-length cDNAs of the *rbcL* and *rbcS* genes were obtained separately from SR and DR, and then deposited in the GenBank. There were no differences between the nucleotide sequences of Rubisco from SR and DR, for either the large (1431 bps) or small (516 bps) subunits (see Supplementary Figure S2). The results suggested that the protein translocation did not result from the amino difference in the sequence and probably depended on a conformational change in the Rubisco itself.

**FIGURE 8:**
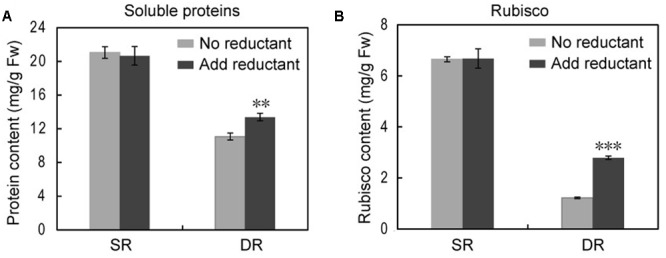
**The effect of the reducing agent ME on the amount of soluble proteins and Rubisco in the swamp ecotype (SR) and desert-dune ecotype (DR) of *Phragmites*.** The soluble protein **(A)** and rubisco **(B)** contents show difference in the absence or presence of reductant (ME). ^∗∗^*P* < 0.01, ^∗∗∗^*P* < 0.001, Student’s *t*-test.

**FIGURE 9:**
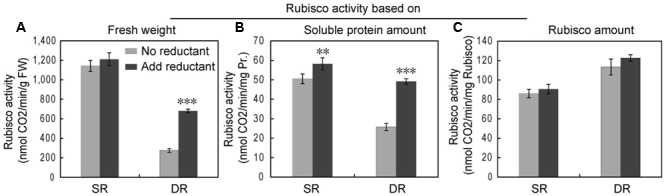
**The effect of the reducing agent ME on Rubisco activity in the SR and desert-dune ecotype (DR) of *Phragmite*.** The relative Rubisco activities per fresh weight **(A)**, soluble protein amount **(B)**, and rubisco amount **(C)** show difference in the absence or presence of reductant (ME). ^∗∗^*P* < 0.01, ^∗∗∗^*P* < 0.001, Student’s *t*-test.

Given the inevitable oxidative stress in a desert environment, we analyzed both the amount and activity of Rubisco in the presence and absence of reductant. The amount of Rubisco was calculated based on the intensity of each protein spot. We found that the extracting buffer with the reductant ME could significantly increase the extracted amounts of soluble proteins in DR, but hardly impacted the proteins in SR (**Figure 8**). Notably, the effect of the reductant on Rubisco was more efficient. In the presence of ME, the amount of soluble proteins and Rubisco increased by 20.8 and 128.7%, respectively, in DR. The results suggested that some soluble proteins, including Rubisco in DR, were released from the insoluble fraction. The activity assay of Rubisco supported this observation. The addition of the reductant remarkably increased Rubisco activity in DR, expressed especially by the amount of soluble proteins (**Figure 9B**). By contrast, the effect of the reductant on Rubisco was limited in SR. The difference in the effect of the reductant between SR and DR was reflected in the differences in the state of Rubisco. Zhao and Zhang (1993) reported that Rubisco from DR contained about 50% of titratable SH groups compared with that from SR, suggesting that the harsh desert environment induced a conformational change in Rubisco. We speculate that the conformational change allowed the enzyme to attach to the thylakoid membrane, resulting in high levels of insoluble proteins in DR. Interestingly, if expressed as the amount of Rubisco, the enzyme had higher activity in DR than in SR (**Figure 9C**). Taken together, the Rubisco distribution between the soluble and insoluble fractions was dynamic and partially regulated by cellular redox status. We propose that the translocation proteome, including Rubisco, may be an unknown plant response to stressful environments.

## Discussion

Many short-term responses of plants to drought and other abiotic stresses have been extensively studied and reported in model plants and cultivated species (Hirayama and Shinozaki, 2010; Krasensky and Jonak, 2012; Singh and Laxmi, 2015). We observed that numerous, highly abundant proteins, which were identified in our previous paper (Cui et al., 2009), were shared in two fractions, but with varying proportions. In this paper, we report an unpredictable response of proteome translocation that occurred in a desert-dune ecotype of *Phragmites*, a long-term response to a complex stress environment. Due to desertification in northwest China, the desert-dune reed (DR) has evolved from its sympatric swamp ecotype (SR) (Zhu et al., 2003). Routinely, DR was frequently subjected to complex stress factors including water deficit, high temperature, and strong radiation and so on. Thus, morphological and physiological alteration was highly significant in DR compared with SR (**Figure 1**). Numerous investigations have been carried out in the field (Zheng et al., 1999; Zhu et al., 2001; Chen et al., 2003, 2007; Gong et al., 2003). However, some perplexing information on the intracellular protein status in the two reed ecotypes remains an intriguing challenge.

Several researchers found that DR possessed a low level of soluble proteins, resulting in a distinct protein profile on SDS-PAGE gels (Wang et al., 1994; Yang et al., 1994). Numerous proteins, including the well-known chloroplast Rubisco protein, disappeared or were only slightly present on the DR map (Ren and Zhang, 1992). In 2009, we developed a three-step method for extracting leaf proteins in SR and DR, and generated the representative proteomes of *Phragmites* (Cui et al., 2009). We revealed that the proportions between soluble and insoluble proteins differed considerably between different ecotypes of *Phragmites*, although both the total amount and pattern of their leaf proteins had high similarity. Therefore, many proteins including Rubisco were not listed in the catalog of differentially expressed proteomes (Cui et al., 2009). To clarify these inconceivable results, in the current study, the soluble and insoluble fractions of reed leaf proteins were carefully separated by repeated grinding and supersonic methods in the respective buffer to alleviate possible cross-contamination. After the extraction steps, remnants were checked using confocal microscopy to ensure consistency in extraction across ecotypes (Supplementary Figure S3). Finally, we confirmed the result that DR exhibits a low level of soluble proteins but a high level of insoluble proteins; the total amount of proteins in DR leaves was similar or less than that in SR, which possibly depended on uncontrollable environmental changes in the field in different sampling years (**Figure 3**). Taking Rubisco as an example, the protein did not disappear in DR as described previously (Ren and Zhang, 1992), but was present in a small amount in the soluble fraction of DR; a large amount of Rubisco was found in the insoluble fraction of DR (**Figures 4**, **7**).

To decipher the alteration in protein proportion between the soluble and insoluble fractions in the two reed ecotypes, high-resolution DIGE gels (24 cm long) were generated from each protein fraction. At least 58 proteins including Rubisco were found and identified; these originally resided in the soluble fraction of SR and are now predominantly present in the insoluble fraction of DR. The identified proteins function in photosynthesis, transport ATPases, protein synthesis, protein folding and stability as well as disease/defense. The majority of the proteins are known chloroplast proteins (**Table 2**). Based on limited available information, we know that the cellular pool of some proteins can be divided into different sub-fractions. For example, HSPs, and small HSPs in particular, were divided into a cytoplasmic and a membranous sub-fraction (Nakamoto and Vigh, 2007). Using immunogold-labeling electron microscopy, Jin et al. (2006) found that 75% of rice RCA was located in the stroma, the remaining 25% was located in the thylakoids; Herbette et al. (2004) found that a glutathione peroxidase-like protein was located in the cytoplasm and cell wall of trichomes and the xylem tissues of tomato; König et al. (2002) observed that 72% of barley 2-Cys Prx attached to the thylakoid membrane. In this paper, we demonstrate that many proteins of field *Phragmites* might be located in two or more sub-cellular/organelle compartments. Through the fractionation proteomic assay, their distribution proportions are quantitatively revealed in **Table 2**. More importantly, we found that the distribution proportions should be dynamic and redox-regulated.

Due to long-term stress in the field, many proteins in DR were transferred into insoluble fraction (**Table 2**). The proteins in insoluble fraction could be defined as membrane-associated proteins (Ryu and Wang, 1996). Therefore, our finding implies that an increased protein association with membranes was developed in DR for coping with harsh environment. The proteins transferred in highest abundance were the large and small subunits of Rubisco, with 52.8 and 52.3% translocation rates, respectively. Because the chloroplast protein is also the most abundant species in reed leaf tissue (Cui et al., 2009), its translocation into the insoluble fraction should be the main reason for the lower level of soluble proteins in DR. Based on current knowledge, no information is available on Rubisco attaching to the thylakoid under stress conditions. After exclusion of the primary sequence difference of Rubisco between SR and DR (Supplementary Figure S2), the translocation response of the protein in DR was ascribed to its conformational change, due to less titratable SH groups in Rubisco purified from DR (Zhao and Zhang, 1993). Further, the Rubisco amount and activity were significantly enhanced in DR when the reductant ME was applied (**Figures 8**, **9**), suggesting that the membrane sequestration of Rubisco was partly reversible and redox-regulated. Certainly, direct evidences are still necessary in order to estimating indirect and non-specific effect of reducing agent on the proteins.

Another protein that translocated remarkably into the insoluble fraction of DR was RCA. Two isoforms of spinach RCAs have been found to attach to the thylakoid in heat-stressed leaves (Rokka et al., 2001). We found at least four membrane-associated forms of RCA with different molecular weights and isoelectric points that were induced in DR, with an average translocation rate of 44.8% (**Table 2**). Of these, the translocation rates of spots 109 and 176 were high, up to 60.2 and 52.1%, respectively (**Table 2** and **Figure 6A**), suggesting different functions among the RCA isoforms in DR adaptation to a stressful environment. In addition to these photosynthetic proteins, those functioning in protein folding and stability also exhibited enhanced membrane association in DR. Various kinds of molecular chaperones including Cpn 60, HSP70, HSP90, and PPI were identified as the group attached to membranes (**Table 2**). Chloroplast chaperonin Cpn 60 in DR displayed the most obvious translocation to the insoluble fraction (**Figure 4Ba**). Although the organelle chaperonins have been assumed to function in protein folding and assisting refolding, in a manner similar to their bacterial homologs, their functional characterization is limited in plants (Wang et al., 2004). Unique structural and functional properties have been indicated in the plastid chaperonins (Levy-Rimler et al., 2002). Chloroplast chaperones including Cpn 60 were implicated in the assembly of the oligomeric enzyme Rubisco (Hemmingsen et al., 1988; Liu et al., 2010). Therefore, it is easy to assume that these proteins co-translocated into the insoluble fraction of DR reflect a structural requirement in response to a long-term adverse environment. In this work, two HSP70 and an HSP90 were also transferred into the insoluble fraction of DR, but there were no small HSP proteins (**Table 2**). Several reports indicate that sHSPs have cytoplasmic and membrane-associated pools. The latter is able to regulate rapidly in response to stresses (Török et al., 2001; Nakamoto and Vigh, 2007), suggesting an altering proportion between the soluble and insoluble fractions. The clues may facilitate future efforts to explore a more complete profile for the stress-induced change in protein distribution. In addition, we found three components that participate in ROS responses, i.e., 2-Cys Prx, peroxiredoxin-2E-2 and thioredoxin M shifted into the insoluble fraction of DR (**Table 2**). Of these, four isoforms of 2-Cys Prx with different isoelectric points displayed a compelling translocation (**Figures 4B**, **6D**). König et al. (2002, 2003) revealed that high salt stress favored oligomerization of barley 2-Cys Prx and triggered membrane attachment, which allowed for detoxification of peroxides at the site of production in the immediate vicinity of the thylakoid membrane. Therefore, the membrane-attached response was helpful for scavenging ROS caused by stressful environmental conditions in photosynthetic tissues.

## Conclusion

Plants subjected to long-term environmental stresses evolve a series of unpredictable responses to regulate metabolism and retain cell vitality. In this study, we report a proteome-wide translocation response, in which proteins located in the soluble fraction of SR surprisingly translocated in the insoluble fraction of DR. In other words, the desert-dune ecotype of *Phragmites* exhibits more ‘bound’ or membrane-associated proteins in comparison to its SR. Given the similarity in the behavior of both the water distribution and the protein distribution in SR and DR, the reinforced ‘bound’ proteins, as well as bound water, emphasize the realistic significance of the membrane association of biomolecules for plant adaptation to complex stress conditions. Further studies about the association of protein with membrane under stress condition are required to elucidate more detailed mechanisms. Protein lipidation (Nadolski and Linder, 2007) and change in membrane lipid composition (Müller and Santarius, 1978) might play a role in the association. We can assume that the membrane association of biomolecules confers advantages in membrane protection and energy efficiency as desert plants thrive in their habitats. Certainly, the results imply that many proteins might possess multiple functions beyond those that are known. Their roles during the adaptation process will also have to be explored in future studies.

## Author Contributions

LL performed most of the experiments and wrote the article. XC performed part of the experiments and data collection. LS performed part of the experiments and provided technical assistance to LL and XC. CW and BF performed data collection and complemented the writing. TQ built the database and complemented the writing. SC conceived the idea, designed the experiments, supervised the experiments and wrote the article.

## Conflict of Interest Statement

The authors declare that the research was conducted in the absence of any commercial or financial relationships that could be construed as a potential conflict of interest.

## Footnotes

1 http://www.geneontology
