# Supplementary material for: A Proteome Translocation Response to Complex Desert Stress Environments in Perennial Phragmites Sympatric Ecotypes with Contrasting Water Availability
Source: Front Plant Sci. 2017 Apr 13;8:511. doi: 10.3389/fpls.2017.00511 (PMC5390029; doi:10.3389/fpls.2017.00511)
Supplement: Supplementary file 1 [file Data_Sheet_1.pdf]

Supporting Information

A proteome translocation response to complex desert stress environments in perennial *Phragmites* sympatric ecotypes with contrasting water availability

Li Li†, Xiaodan Chen†, Lu Shi†, Chuanjing Wang, Bing Fu, Tianhang Qiu, Suxia Cui*

College of Life Sciences, Capital Normal University, Beijing 100048, P. R. China

*Corresponding author

E-mail: sxcui@cnu.edu.cn

†Li Li, Xiaodan Chen, Lu Shi, these authors contributed equally to this work.

Table of contents

Supplementary Materials and Methods

Table S1, S2, S3, S4, S5

Figure S1, S2, S3

Supplementary Materials and Methods

Sampling site

Two ecotypes of *Phragmites communis*, i.e. swamp reed (SR) and desert-dune reed (DR), inhabit in southern margin of the Badanjilin desert in Northwest China. SR naturally grows in a nameless rivulet; DR grows on natural sand dunes with height of 2-5 meters. The two ecotypes of *Phragmites* are typical sympatric populations distributing a narrow area with about 6.5 km^2 (39°31'–58'N, 100°4'–36'E; elevation 1300 m). The mean annual precipitation of the sampling site is only 118 mm, the annual potential evaporation is 2392 mm, and with large daily fluctuation of air temperature (data from local Meteorological Bureau). In this study, three biological replicates for each reed ecotype were collected as shown in the diagram (Fig S1).

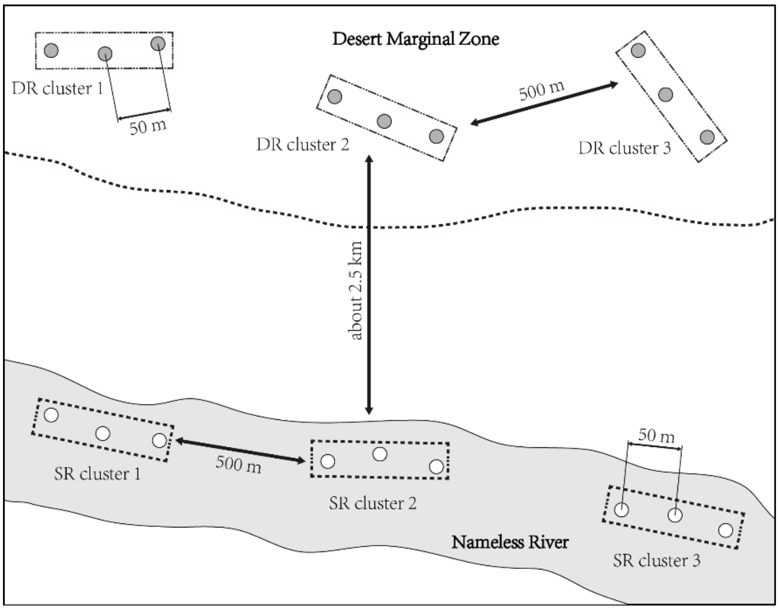

Fig S1 Position of sampling and constitution of three biological replicates.

Protein Cydye Labeling and DIGE design

Proteins from both soluble fraction (I) and insoluble fraction (II) were extracted from DR and SR, respectively. Total 12 protein samples, including three biological replicates for each ecotypes, were labeled reciprocally with Cy3 and Cy5 (Table S1) according to the method described by Cui et al[1]. Briefly, protein samples were suspended in lysis buffer (7 M urea, 2 M thiourea, and 4% CHAPS) and adjusting the pH to 8.5; 50 μg of proteins were mixed with 300 pmol of CyDye (GE Healthcare) and incubated on ice in the dark for 30 min. The labelling reaction was stopped by the addition of lysine. An internal standard was generated by pooling equal amounts of proteins from each sample, and then labelled with Cy2.

Table S1 Labeled samples for the DIGE experiment. Three biological replicates were marked in Arabic numbers. Two fractions of proteins were marked in Roman numbers.

Gels	Labeled by DIGE-specific		
	Cy3	Cy5	Cy2
Gel1	SR1-I	DR1-I	12 samples mixed
Gel2	DR1-II	SR1-II	
Gel3	DR2-I	SR2-I	
Gel4	SR2-II	DR2-II	
Gel5	DR3-I	DR3-II	
Gel6	SR3-I	SR3-II	

2-DE and In-gel Tryptic Digestion

Traditional 2-DE gels stained with Coomassie brilliant blue (CBB) were prepared for protein identification[2]. For iso-electrofocusing (IEF), 900 μg of proteins were re-suspended in IEF solution containing 7 M urea, 2 M thiourea, 4% CHAPS, 40mM DTT and 0.5% (v/v) immobilized pH gradient buffer (pH 3-10). After centrifugation at 9,000 × g for 3 min, the supernatant was applied onto a linear immobilized pH gradient strip (pH 4-7, 24 cm). For second dimension, the strips were incubated in an equilibration buffer (6 M urea, 30% (v/v) glycerol and 2% SDS in 0.05 M Tris-HCl buffer, pH 8.8) containing 15 mm DTT for 15 min as the first step, then replaced by 2.5% iodoacetamide as the second step. Following SDS-PAGE, proteins were detected by CBB R-250. The spots of interest were sampled and submitted to in-gel digestion with trypsin. Trypsin digestion was performed overnight at 37°C and stopped by adding 5% formic acid.

Mass Spectrometry, and Database Search

Extracted peptides were analyzed by MALDI TOF/TOF MS (Ultraflex III, Bruker Daltonics, Bremen, Germany) as previously described[1]. In brief, samples were spotted onto a MALDI target plate using cyano-4-hydroxycinnamic acid matrix. Mass data acquisitions were piloted automatically by an autoXecute method within the FlexControl software v3.0. In MS mode, spectra were acquired in a mass range m/z 800–4500 by summing up 2000 laser shots with an acceleration of 23 kV. The MS spectra were externally calibrated using PeptideCalibStandard II (Bruker Daltonics) (1046.542, 1296.685, 1347.735, 1619.822, 2093.086, 2465.198, and 3147.471), resulting in mass errors of <50 ppm. The MS peaks were detected with a minimum signal/noise (S/N) ratio >20 and cluster area S/N threshold >25 without smoothening and raw spectrum filtering. Peptide precursor ions corresponding to contaminants including keratin and the trypsin autolytic products were excluded in a mass tolerance of 0.2 Da. For acquiring MS/MS spectra, the filtered precursor ions with a user-defined threshold (S/N ratio >50) were selected. Fragmentation of precursor ions was performed using the LIFT positive mode. MS/MS spectra were accumulated from 4000 laser shots. The MS/MS peaks were detected on a minimum S/N ratio >3 and a cluster area S/N threshold >15 with smoothing. Mass spectra were piloted using FlexAnalysis software.

The obtained MS and MS/MS spectra per spot were combined, and submitted to MASCOT search engine by Biotools 3.1 (Bruker Daltonics). Parameters selected included: MS/MS Ion Search, the NCBInr database of green plants 20160731

(91579380 sequences; 33741759339 residues), trypsin of the digestion enzyme, up to one missed cleavage site, parent ion mass tolerance at 100 ppm, MS/MS mass tolerance of 0.5 Da, carbamidomethylation of cysteine (global modification), and methionine oxidation (variable modification). The probability score (95% confidence level) calculated by the software, and a matching of at least 2 peptides was used as a criterion for correct identification. The MS/MS data set for all proteins are provided in Table S2 file.

Gene Cloning and Sequencing for Large and Small Subunits of Rubisco

To obtain the full-length cDNA of the rbcL and rbcS genes, DOP-PCR (degenerated oligonucleotideo-primed-PCR), 3’-RACE (rapid-amplification of cDNA ends) and 5’-TAIL-PCR (Thermal asymmetric interlaced polymerase chain reaction) were performed by using the double-stranded cDNA of SR and DR as a template, respectively. The primer pairs used for DOP-PCR for rbcL gene were the sense primer 5’-TTTCACAAGCTGCGGCTAGTTC-3’ and the antisense primer 5’-TTCGTTA CAAAGGCCGATGCTA-3’ and for rbcS gene were the sense primer 5’-CCT TCCAGGGGCTCA-3’, and the antisense primer 5’-GGGCTTGTAGGCGATG-3’. In the 3’-RACE, double-stranded cDNA was acquired using primer 5’-GACTCGA GTCGACATCGATTTTTTTTTTTTTTTTT-3’, and then the common sense primer 5’-TCTTGCTCGTGAAGGTAATGAA-3’, the antisense primers 5’-GACTCGAGT CGACATCG-3’ (rbcL) and 5’-GCATTCGTCCGTATCAT-3’ (rbcS) were used for rapid-amplification of 3’-cDNA ends, respectively. 5’-cDNA ends were determined by the TAIL-PCR method as antecedently described[3, 4]. The three PCR reactions were included as Table S3, and it was necessary to further distinguish between the specific and non-specific products. The sequences of the specific primers and four arbitrary degenerate (AD) primers are given in Table S4, with the following identities: LSP1-LSP3 were specific primers for rbcL, SSP1-SSP3 were specific primers for rbcS and AD1-AD4 were common AD primers for rbcL and rbcS. The primer concentrations in the primary and secondary TAIL-PCR reactions were 0.6 pmol/μl and 1.3 pmol/μl for the right border-specific and arbitrary primers, respectively. In the tertiary TAIL

reactions the concentrations of both primers were 0.6 pmol/μl.

The resulting PCR products were ligated into vector TA, cloned, and sequenced. Every sequence was confirmed by examining both strands; in addition, at least two bacterial clones obtained by TA cloning were examined. By assembling the sequences of 3', 5' and the core partial sequences on ContigExpress (Vector NTI Advance11.5.1), the full-length cDNA sequence of rbcL and rbcS gene was deduced. The nucleotide sequence data for rbcL and rbcS, from SR and DR, have been deposited in the GenBank nucleotide sequence databases under accession no. KF697233, KF697234, KF697235 and KF697236, respectively. Finally, the sequence alignments between SR and DR were determined using AlignX (Vector NTI Advance11.5.1) (Figure S2).

Table S3. Thermal conditions and cycle settings used for TAIL-PCR.

Reaction	Thermal settings	Cycle No.
Primary	92°C 2min，95°C 1min	1
	95°C 15s，63°C 1min，72 °C 2min	5
	94 °C 15s，30 °C 3min，to 72 °C at 0. 2 °C/ s，72°C 2min	1
	94°C 5s，95°C 1min，72°C 2min	10
	94°C 5s，63°C 1min，72°C 2min	
	94°C 5s，58°C 1min，72°C 2min	12
	94°C 5s，44°C 1min，72°C 2min	
	72°C 7min，4°C forever	1
Secondary	94°C 5s，63°C 1min，72°C 2min	
	94°C 5s，58°C 1min，72°C 2min	10
	94°C 5s，44°C 1min，72°C 2min	
	72°C 7min，4°C forever	1
Tertiary	94°C 5s，63°C 1min，72°C 2min	
	94°C 5s，58°C 1min，72°C 2min	20
	94°C 5s，44°C 1min，72°C 2min	
	72°C 7min， 4°C forever	1

Table S4. Primers used in TAIL-PCR.

Primer name	Primer sequence
LSP1	5‘-TTGACCCTCGTCCCCAGGAACGGGCTCG-3’
LSP2	5’-AGGCGGACCTTGGAAAGTTTTTGAATAA-3’
LSP3	5’-CTTCAGCACAAAAGACAAAACGGT-3’
SSP1	5’-GTGGACTTGAGCCCCTGGAA-3’
SSP2	5’-ATCCTGCCGCCATTGCTGAC-3’
SSP3	5’-GGTAGACGAATCCGACCTTGCTGAA-3’
AD1	5’-NGACGASWGANAWGAA-3’
AD2	5’-NGTCGASWGANAWGAA-3’
AD3	5’-AGWGNAGWANCAWAGG-3’
AD4	5’-WGTGNAGWANCANAGA-3’

Figure S2.

A. Rubisco large subunit CDS

```
SR-P.communis    ATGTCACCACAGACAGAAACTAAAGCAAGTGTTGGATTTAAAGCCGGTGTTAAGGATTATAAATTGACTT     70
DR-P.communis    ATGTCACCACAGACAGAAACTAAAGCAAGTGTTGGATTTAAAGCCGGTGTTAAGGATTATAAATTGACTT     70
O.sativa         ATGTCACCACAAACAGAAACTAAAGCAAGTGTTGGATTTAAAGCTGGTGTTAAGGATTATAAATTGACTT     70
Z.mays           ATGTCACCACAAACAGAAACTAAAGCAAGTGTTGGATTTAAAGCTGGTGTTAAGGATTATAAATTGACTT     70
A.thaliana       ATGTCACCACAAACAGAGACTAAAGCAAGTGTTGGGTTCAAAGCTGGTGTTAAAGAGTATAAATTGACTT     70

SR-P.communis    ACTACACCCCGGAGTACGAAACCAAGGATACTGATATCTTGGCAGCATTCCGAGTAACTCCTCAGCCCGG    140
DR-P.communis    ACTACACCCCGGAGTACGAAACCAAGGATACTGATATCTTGGCAGCATTCCGAGTAACTCCTCAGCCCGG    140
O.sativa         ACTACACCCCGGAGTACGAAACCAAGGACACTGATATCTTGGCAGCATTCCGAGTAACTCCTCAGCCGGG    140
Z.mays           ACTACACCCCGGAGTACGAAACCAAGGATACTGATATCTTGGCAGCATTCCGAGTAACTCCTCAGCTCGG    140
A.thaliana       ACTATACTCCTGAATATGAAACCAAGGATACTGATATCTTGGCAGCATTCCGAGTAACTCCTCAACCTGG    140

SR-P.communis    GGTTCCGGCTGAAGAAGCAGGGGCTGCAGTAGCTGCGGAATCTTCTACTGGTACATGGACAACTGTTTGG    210
DR-P.communis    GGTTCCGGCTGAAGAAGCAGGGGCTGCAGTAGCTGCGGAATCTTCTACTGGTACATGGACAACTGTTTGG    210
O.sativa         GGTTCCGCCCGAAGAAGCAGGGGCTGCAGTAGCTGCCGAATCTTCTACTGGTACATGGACAACTGTTTGG    210
Z.mays           GGTTCCGCCTGAAGAAGCAGGAGCTGCAGTAGCTGCGGAATCTTCTACTGGTACATGGACAACTGTTTGG    210
A.thaliana       AGTTCCACCTGAAGAAGCAGGGGCTGCGGTAGCTGCTGAATCTTCTACTGGTACATGGACAACTGTGTGG    210

SR-P.communis    ACTGATGGACTTACCAGTCTTGATCGTTACAAAGGCCGATGCTATCACATCGAGCCCGTTCCTGGGGACG    280
DR-P.communis    ACTGATGGACTTACCAGTCTTGATCGTTACAAAGGCCGATGCTATCACATCGAGCCCGTTCCTGGGGACG    280
O.sativa         ACTGATGGACTTACCAGTCTTGATCGTTACAAAGGCCGATGCTATCACATCGAGCCCGTTGTTGGGGAGG    280
Z.mays           ACTGATGGACTTACCAGTCTTGATCGTTACAAAGGACGATGCTATCACATCGAGCCCGTTCCTGGGGACC    280
A.thaliana       ACCGATGGGCTTACCAGCCTTGATCGTTACAAAGGACGATGCTACCACATCGAGCCCGTTCCAGGAGAAG    280

SR-P.communis    AGGGTCAATATATCTGTTATGTAGCTTATCCATTAGACCTATTTGAAGAGGGTTCTGTTACTAACATGTT    350
DR-P.communis    AGGGTCAATATATCTGTTATGTAGCTTATCCATTAGACCTATTTGAAGAGGGTTCTGTTACTAACATGTT    350
O.sativa         ATAATCAATATATCGCTTATGTAGCTTATCCATTAGACCTATTTGAAGAGGGTTCTGTTACTAACATGTT    350
Z.mays           CAGATCAATATATCTGTTATGTAGCTTATCCATTAGACCTATTTGAAGAGGGTTCTGTTACTAACATGTT    350
A.thaliana       AAACTCAATTTATTGCGTATGTAGCTTATCCCTTAGACCTTTTTGAAGAAGGTTCGGTTACTAACATGTT    350

SR-P.communis    TACTTCCATTGTAGGTAACGTATTTGGTTTCAAAGCCCTACGCGCTCTACGTTTGGAGGATCTACGAATT    420
DR-P.communis    TACTTCCATTGTAGGTAACGTATTTGGTTTCAAAGCCCTACGCGCTCTACGTTTGGAGGATCTACGAATT    420
O.sativa         TACTTCCATTGTGGGTAACGTATTTGGTTTCAAAGCCCTACGCGCTCTACGTCTGGAGGATCTGCGAATT    420
Z.mays           TACTTCCATTGTGGGTAACGTATTTGGTTTCAAAGCCTTACGCGCTCTACGTTTGGAGGATCTACGAATT    420
A.thaliana       TACCTCGATTGTGGGTAATGTATTTGGGTTCAAAGCCCTGGCTGCTCTACGTCTAGAGGATCTGCGAATC    420

SR-P.communis    CCTCCTACTTATTCAAAAACTTTCCAAGGTCCGCCTCATGGTATCCAAGTTGAAAGGGATAAGTTGAACA    490
DR-P.communis    CCTCCTACTTATTCAAAAACTTTCCAAGGTCCGCCTCATGGTATCCAAGTTGAAAGGGATAAGTTGAACA    490
O.sativa         CCCCCTACTTATTCAAAAACTTTCCAAGGTCCGCCTCATGGTATCCAAGTTGAAAGGGATAAGTTGAACA    490
Z.mays           CCCCCTGCTTATTCAAAAACTTTCCAAGGTCCGCCTCACGGTATCCAAGTTGAAAGGGATAAGTTGAACA    490
A.thaliana       CCTCCTGCTTATACTAAAACTTTCCAAGGACCACCTCATGGTATCCAAGTTGAAAGAGATAAATTGAACA    490

SR-P.communis    AGTATGGCCGTCCTTTATTGGGATGTACTATTAAACCAAAATTGGGATTATCCGCAAAAAATTACGGTAG    560
DR-P.communis    AGTATGGCCGTCCTTTATTGGGATGTACTATTAAACCAAAATTGGGATTATCCGCAAAAAATTACGGTAG    560
O.sativa         AATACGGTCGTCCTTTATTGGGATGTACTATTAAACCAAAATTGGGATTATCTGCAAAAAATTATGGTAG    560
Z.mays           AGTACGGTCGTCCTTTATTGGGATGTACTATTAAACCAAAATTGGGATTATCCGCAAAAAATTACGGTAG    560
A.thaliana       AGTATGGACGTCCCCTATTAGGATGTACTATTAAACCAAAATTGGGGTTATCCGCGAAAAACTATGGTAG    560

SR-P.communis    AGCTTGTTATGAGTGTCTACGCGGTGGACTTGATTTTACCAAAGATGATGAAAACGTAAACTCACAACCA    630
DR-P.communis    AGCTTGTTATGAGTGTCTACGCGGTGGACTTGATTTTACCAAAGATGATGAAAACGTAAACTCACAACCA    630
O.sativa         AGCATGTTATGAGTGTCTACGCGGTGGACTTGATTTTACCAAAGATGATGAAAACGTAAACTCACAACCA    630
Z.mays           AGCGTGTTATGAGTGTCTACGCGGTGGACTTGATTTTACCAAAGATGATGAAAACGTAAACTCACAACCA    630
A.thaliana       AGCAGTTTATGAATGTCTACGTGGTGGACTTGATTTTACCAAAGATGATGAGAATGTGAACTCCCAACCA    630

SR-P.communis    TTTATGCGCTGGAGAGACCGTTTTGTCTTTTGTGCTGAAGCAATTTATAAATCACAGGCCGAAACCGGTG    700
DR-P.communis    TTTATGCGCTGGAGAGACCGTTTTGTCTTTTGTGCTGAAGCAATTTATAAATCACAGGCCGAAACCGGTG    700
O.sativa         TTTATGCGTTGGAGGGACCGTTTTGTCTTTTGTGCCGAAGCTATTTATAAATCACAGGCCGAAACCGGTG    700
Z.mays           TTTATGCGCTGGAGAGACCGTTTCGTCTTTTGTGCCGAAGCAATTTATAAAGCACAAGCCGAAACTGGTG    700
A.thaliana       TTTATGCGTTGGAGAGACCGTTTCTTATTTTGTGCCGAAGCTATTTATAAATCACAGGCTGAAACAGGTG    700

SR-P.communis    AAATCAAGGGGCATTACTTGAATGCGACTGCAGGTACATGCGAAGAAATGATTAAGAGAGCTGTATTTGC    770
DR-P.communis    AAATCAAGGGGCATTACTTGAATGCGACTGCAGGTACATGCGAAGAAATGATTAAGAGAGCTGTATTTGC    770
O.sativa         AAATTAAGGGGCATTACTTGAATGCGACTGCAGGTACATGCGAAGAAATGATTAAAAGAGCTGTATTTGC    770
Z.mays           AAATCAAGGGGCATTACTTGAATGCGACTGCAGGTACATGCGAAGAAATGATTAAGAGAGCTGTATTTGC    770
A.thaliana       AAATCAAAGGGCATTATTTGAATGCTACTGCGGGTACATGCGAAGAAATGATCAAAAGAGCTGTATTTGC    770

SR-P.communis    GAGGGAATTAGGGGTTCCTATTGTAATGCATGACTACTTAACTGGAGGATTCACCGCAAATACTACTTTG    840
DR-P.communis    GAGGGAATTAGGGGTTCCTATTGTAATGCATGACTACTTAACTGGAGGATTCACCGCAAATACTACTTTG    840
O.sativa         GAGGGAATTAGGGGTTCCTATTGTAATGCATGACTACTTAACCGGGGGGTTCACCGCAAATACTAGTTTG    840
Z.mays           AAGGGAATTAGGGGTTCCTATTGTAATGCATGACTACTTAACAGGAGGATTCACCGCAAATACTACTTTG    840
A.thaliana       CAGAGAATTGGGAGTTCCTATCGTAATGCATGACTACTTAACAGGGGGATTCACCGCAAATACTAGTTTG    840
```

```
SR-P.communis    GCTCATTATTGCCGCGACAACGGCCTACTTCTTCACATTCACCGAGCAATGCATGCAGTTATTGATAGAC    910
DR-P.communis    GCTCATTATTGCCGCGACAACGGCCTACTTCTTCACATTCACCGAGCAATGCATGCAGTTATTGATAGAC    910
O.sativa         GCTCATTATTGCCGCGACAACGGCCTACTTCTTCACATTCACCGAGCAATGCATGCAGTTATTGATAGAC    910
Z.mays           TCTCATTATTGCCGCGACAACGGCCTACTTCTTCACATTCACCGAGCAATGCATGCAGTTATTGATAGAC    910
A.thaliana       TCTCATTATTGCCGAGATAATGGCCTACTTCTTCACATCCACCGTGCAATGCACGCTGTTATTGATAGAC    910

SR-P.communis    AGAAAAATCATGGTATGCATTTCCGTGTATTAGCTAAAGCATTGCGTATGTCTGGGGGAGATCATATCCA    980
DR-P.communis    AGAAAAATCATGGTATGCATTTCCGTGTATTAGCTAAAGCATTGCGTATGTCTGGGGGAGATCATATCCA    980
O.sativa         AGAAAAATCATGGTATGCATTTCCGTGTATTAGCTAAAGCATTGCGTATGTCTGGGGGAGATCATATCCA    980
Z.mays           AGAAAAATCATGGTATGCATTTCCGTGTATTAGCTAAAGCATTGCGTATGTCGGGGGGAGATCATATCCA    980
A.thaliana       AGAAGAATCATGGTATGCACTTCCGTGTACTAGCTAAAGCTTTACGTCTATCTGGTGGAGATCATATTCA    980

SR-P.communis    CGCCGGTACAGTAGTAGGTAAGTTAGAAGGGGAACGCGAAATGACTTTAGGTTTTGTTGATTTATTGCGC   1050
DR-P.communis    CGCCGGTACAGTAGTAGGTAAGTTAGAAGGGGAACGCGAAATGACTTTAGGTTTTGTTGATTTATTGCGC   1050
O.sativa         CGCTGGTACAGTAGTAGGTAAGTTAGAAGGGGAACGCGAAATGACTTTAGGTTTTGTTGATTTATTGCGC   1050
Z.mays           CTCCGGTACAGTAGTAGGTAAGTTAGAAGGGGAACGCGAAATAACTTTAGGTTTTGTTGATTTATTGCGC   1050
A.thaliana       CGCGGGTACAGTAGTAGGTAAACTTGAAGGAGACAGGGAGTCAACTTTGGGCTTTGTTGATTTACTGCGC   1050

SR-P.communis    GATGATTTTATTGAAAAAGACCGTGCTCGCGGTATCTTTTTCACTCAGGACTGGGTATCCATGCCAGGTG   1120
DR-P.communis    GATGATTTTATTGAAAAAGACCGTGCTCGCGGTATCTTTTTCACTCAGGACTGGGTATCCATGCCAGGTG   1120
O.sativa         GATGATTTTATTGAAAAAGATCGTGCTCGCGGTATCTTTTTCACTCAGGACTGGGTATCCATGCCAGGTG   1120
Z.mays           GATGATTTTATTGAAAAAGATCGTTCTCGCGGTATCTTTTTCACTCAGGACTGGGTATCCATGCCAGGTG   1120
A.thaliana       GATGATTATGTTGAAAAAGATCGAAGCCGCGGTATCTTTTTCACTCAAGATTGGGTCTCACTACCTGGTG   1120

SR-P.communis    TTATACCGGTGGCTTCAGGGGGTATTCATGTTTGGCATATGCCAGCTCTGACCGAAATCTTTGGAGACGA   1190
DR-P.communis    TTATACCGGTGGCTTCAGGGGGTATTCATGTTTGGCATATGCCAGCTCTGACCGAAATCTTTGGAGACGA   1190
O.sativa         TTATACCGGTGGCTTCAGGGGGTATTCATGTTTGGCATATGCCAGCTCTGACCGAAATCTTTGGAGATGA   1190
Z.mays           TTATACCGGTGGCTTCTGGGGGTATTCATGTTTGGCATATGCCAGCTCTGACCGAAATCTTTGGAGATGA   1190
A.thaliana       TTCTGCCTGTGGCTTCAGGGGGTATTCACGTTTGGCATATGCCTGCTTTGACCGAGATCTTTGGAGATGA   1190

SR-P.communis    TTCCGTATTACAATTTGGTGGAGGAACTTTAGGACACCCTTGGGGGAATGCACCTGGTGCAGCAGCTAAT   1260
DR-P.communis    TTCCGTATTACAATTTGGTGGAGGAACTTTAGGACACCCTTGGGGGAATGCACCTGGTGCAGCAGCTAAT   1260
O.sativa         TTCTGTATTGCAATTTGGTGGAGGAACTTTAGGACATCCTTGGGGTAATGCACCTGGTGCAGCAGCTAAT   1260
Z.mays           TTCCGTATTACAATTTGGTGGAGGAACTTTAGGACATCCTTGGGGAAATGCACCTGGTGCAGCAGCTAAT   1260
A.thaliana       TTCTGTACTACAATTCGGTGGAGGAACTTTAGGCCACCCTTGGGGAAATGCACCGGGTGCCGTAGCCAAC   1260

SR-P.communis    CGGGTGGCTTTAGAAGCCTGTGTACAAGCTCGTAACGAAGGGCGCGATCTTGCTCGTGAAGGTAATGAAA   1330
DR-P.communis    CGGGTGGCTTTAGAAGCCTGTGTACAAGCTCGTAACGAAGGGCGCGATCTTGCTCGTGAAGGTAATGAAA   1330
O.sativa         CGGGTGGCTTTAGAAGCCTGTGTACAAGCTCGTAACGAAGGGCGCGATCTTGCTCGTGAAGGTAATGAAA   1330
Z.mays           CGTGTGGCTTTAGAAGCCTGTGTACAAGCTCGTAACGAAGGGCGCGATCTTGCTCGTGAAGGTAATGAAA   1330
A.thaliana       CGAGTAGCTCTGGAAGCATGTGTACAAGCTCGTAATGAGGGACGTGATCTTGCAGTCGAGGGTAATGAAA   1330

SR-P.communis    TTATCCGACAAGCTTGCAAATGGAGTCCTGAACTAGCCGCAGCTTGTGAAATATGGAAGGCGATCAAATT   1400
DR-P.communis    TTATCCGACAAGCTTGCAAATGGAGTCCTGAACTAGCCGCAGCTTGTGAAATATGGAAGGCGATCAAATT   1400
O.sativa         TTATCCGATCAGCTTGCAAATGGAGTCCTGAACTAGCCGCAGCTTGTGAAATATGGAAAGCGATCAAATT   1400
Z.mays           TTATCAAAGCAGCTTGCAAATGGAGTGCTGAACTAGCCGCAGCTTGTGAAATATGGAAGGAGATCAAATT   1400
A.thaliana       TTATCCGTGAAGCTTGCAAATGGAGTCCTGAACTAGCTGCTGCTTGTGAAGTATGGAAAGAGATCACATT   1400

SR-P.communis    CGAG...TTCGCGCCGGTAGATACCATCGATTAA.........                              1431
DR-P.communis    CGAG...TTCGCGCCGGTAGATACCATCGATTAA.........                              1431
O.sativa         CGAG...TTCGAGCCGGTAGATAAACTAGATAGCTAG......                              1434
Z.mays           TGATGGTTTCAAAGCGATGGATACCATATAA............                              1431
A.thaliana       TAAC...TTCCCAACCATCGATAAATTAGATGGCCAAGAGTAG                              1440
```

B. Rubisco large subunit (amino acid sequence)

```
SR-P.communis     MSPQTETKASVGFKAGVKDYKLTYYTPEYETKDTDILAAFRVTPQPGVPAEEAGAAVAAESSTGTWTTVW     70
DR-P.communis     MSPQTETKASVGFKAGVKDYKLTYYTPEYETKDTDILAAFRVTPQPGVPAEEAGAAVAAESSTGTWTTVW     70
O.sativa          MSPQTETKASVGFKAGVKDYKLTYYTPEYETKDTDILAAFRVTPQPGVPPEEAGAAVAAESSTGTWTTVW     70
Z.mays            MSPQTETKASVGFKAGVKDYKLTYYTPEYETKDTDILAAFRVTPQLGVPPEEAGAAVAAESSTGTWTTVW     70
A.thaliana        MSPQTETKASVGFKAGVKEYKLTYYTPEYETKDTDILAAFRVTPQPGVPPEEAGAAVAAESSTGTWTTVW     70

SR-P.communis     TDGLTSLDRYKGRCYHIEPVPGDEGQYICYVAYPLDLFEEGSVTNMFTSIVGNVFGFKALRALRLEDLRI    140
DR-P.communis     TDGLTSLDRYKGRCYHIEPVPGDEGQYICYVAYPLDLFEEGSVTNMFTSIVGNVFGFKALRALRLEDLRI    140
O.sativa          TDGLTSLDRYKGRCYHIEPVVGEDNQYIAYVAYPLDLFEEGSVTNMFTSIVGNVFGFKALRALRLEDLRI    140
Z.mays            TDGLTSLDRYKGRCYHIEPVPGDPDQYICYVAYPLDLFEEGSVTNMFTSIVGNVFGFKALRALRLEDLRI    140
A.thaliana        TDGLTSLDRYKGRCYHIEPVPGEETQFIAYVAYPLDLFEEGSVTNMFTSIVGNVFGFKALAALRLEDLRI    140

SR-P.communis     PPTYSKTFQGPPHGIQVERDKLNKYGRPLLGCTIKPKLGLSAKNYGRACYECLRGGLDFTKDDENVNSQP    210
DR-P.communis     PPTYSKTFQGPPHGIQVERDKLNKYGRPLLGCTIKPKLGLSAKNYGRACYECLRGGLDFTKDDENVNSQP    210
O.sativa          PPTYSKTFQGPPHGIQVERDKLNKYGRPLLGCTIKPKLGLSAKNYGRACYECLRGGLDFTKDDENVNSQP    210
Z.mays            PPAYSKTFQGPPHGIQVERDKLNKYGRPLLGCTIKPKLGLSAKNYGRACYECLRGGLDFTKDDENVNSQP    210
A.thaliana        PPAYTKTFQGPPHGIQVERDKLNKYGRPLLGCTIKPKLGLSAKNYGRAVYECLRGGLDFTKDDENVNSQP    210

SR-P.communis     FMRWRDRFVFCAEAIYKSQAETGEIKGHYLNATAGTCEEMIKRAVFARELGVPIVMHDYLTGGFTANTTL    280
DR-P.communis     FMRWRDRFVFCAEAIYKSQAETGEIKGHYLNATAGTCEEMIKRAVFARELGVPIVMHDYLTGGFTANTTL    280
O.sativa          FMRWRDRFVFCAEAIYKSQAETGEIKGHYLNATAGTCEEMIKRAVFARELGVPIVMHDYLTGGFTANTSL    280
Z.mays            FMRWRDRFVFCAEAIYKAQAETGEIKGHYLNATAGTCEEMIKRAVFARELGVPIVMHDYLTGGFTANTTL    280
A.thaliana        FMRWRDRFLFCAEAIYKSQAETGEIKGHYLNATAGTCEEMIKRAVFARELGVPIVMHDYLTGGFTANTSL    280

SR-P.communis     AHYCRDNGLLLHIHRAMHAVIDRQKNHGMHFRVLAKALRMSGGDHIHAGTVVGKLEGEREMTLGFVDLLR    350
DR-P.communis     AHYCRDNGLLLHIHRAMHAVIDRQKNHGMHFRVLAKALRMSGGDHIHAGTVVGKLEGEREMTLGFVDLLR    350
O.sativa          AHYCRDNGLLLHIHRAMHAVIDRQKNHGMHFRVLAKALRMSGGDHIHAGTVVGKLEGEREMTLGFVDLLR    350
Z.mays            SHYCRDNGLLLHIHRAMHAVIDRQKNHGMHFRVLAKALRMSGGDHIHSGTVVGKLEGEREITLGFVDLLR    350
A.thaliana        SHYCRDNGLLLHIHRAMHAVIDRQKNHGMHFRVLAKALRLSGGDHIHAGTVVGKLEGDRESTLGFVDLLR    350

SR-P.communis     DDFIEKDRARGIFFTQDWVSMPGVIPVASGGIHVWHMPALTEIFGDDSVLQFGGGTLGHPWGNAPGAAAN    420
DR-P.communis     DDFIEKDRARGIFFTQDWVSMPGVIPVASGGIHVWHMPALTEIFGDDSVLQFGGGTLGHPWGNAPGAAAN    420
O.sativa          DDFIEKDRARGIFFTQDWVSMPGVIPVASGGIHVWHMPALTEIFGDDSVLQFGGGTLGHPWGNAPGAAAN    420
Z.mays            DDFIEKDRSRGIFFTQDWVSMPGVIPVASGGIHVWHMPALTEIFGDDSVLQFGGGTLGHPWGNAPGAAAN    420
A.thaliana        DDYVEKDRSRGIFFTQDWVSLPGVLPVASGGIHVWHMPALTEIFGDDSVLQFGGGTLGHPWGNAPGAVAN    420

SR-P.communis     RVALEACVQARNEGRDLAREGNEIIRQACKWSPELAAACEIWKAIKFE.FAPVDTID...              476
DR-P.communis     RVALEACVQARNEGRDLAREGNEIIRQACKWSPELAAACEIWKAIKFE.FAPVDTID...              476
O.sativa          RVALEACVQARNEGRDLAREGNEIIRSACKWSPELAAACEIWKAIKFE.FEPVDKLDS..              477
Z.mays            RVALEACVQARNEGRDLAREGNEIIKAACKWSAELAAACEIWKEIKFDGFKAMDTI....              476
A.thaliana        RVALEACVQARNEGRDLAVEGNEIIREACKWSPELAAACEVWKEITFN.FPTIDKLDGQE              479
```

C. Rubisco small subunit CDS

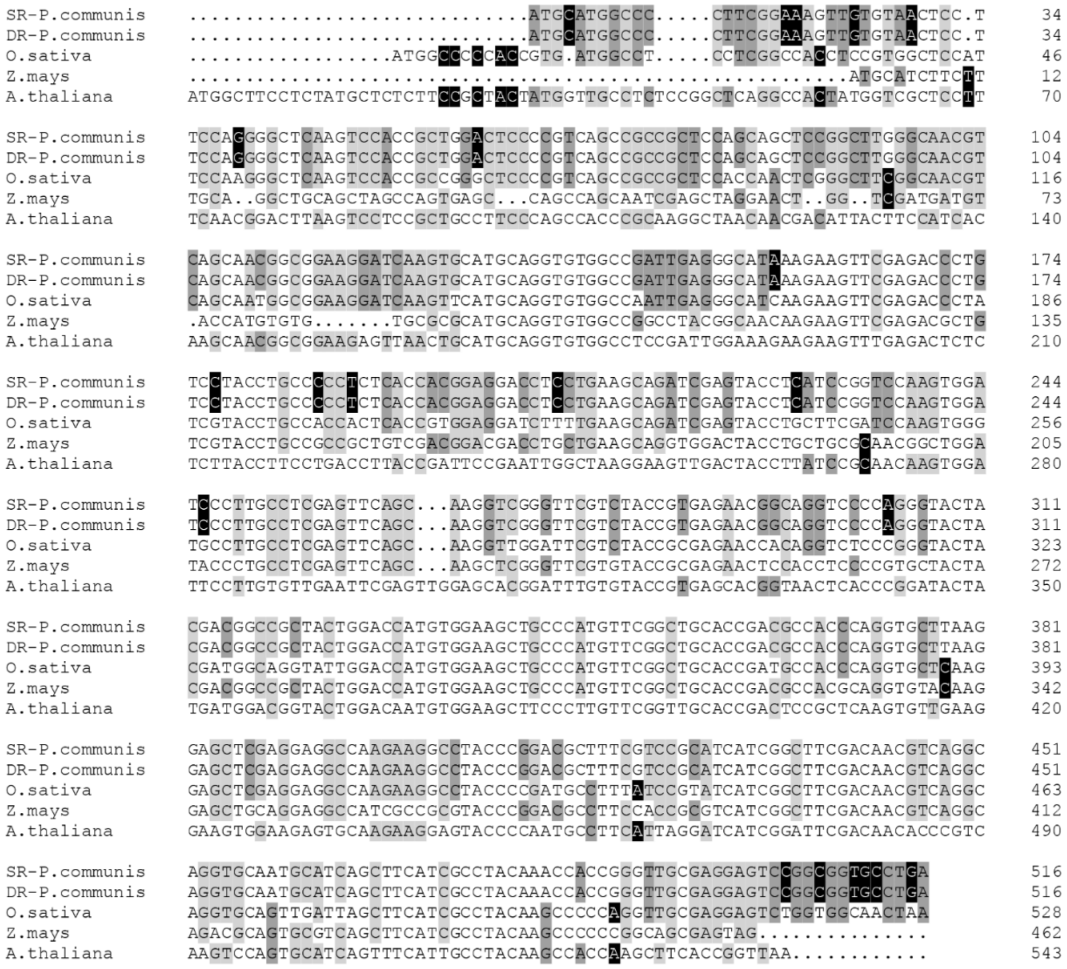

D. Rubisco small subunit (amino acid sequence)

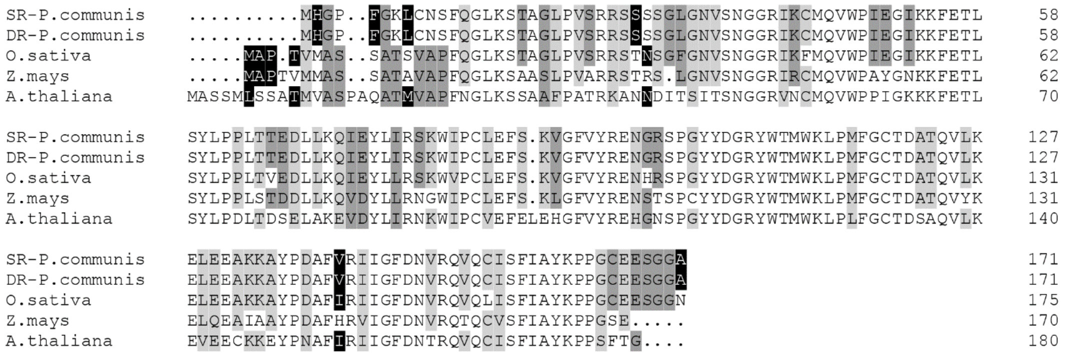

Figure S2 Sequence alignment of both cDNAs and proteins of Rubisco from different plant species. They are *P. communis* (common reed), SR (swamp reed); DR (desert-dune reed), *O. sativa* (rice), *Z. mays* (maize) and *A. thaliana* (arabidopsis). The conserved sequences between five species are marked by different grayscales. White means 100%; Light grey < 75%; Deep grey < 50%; Black <25%.

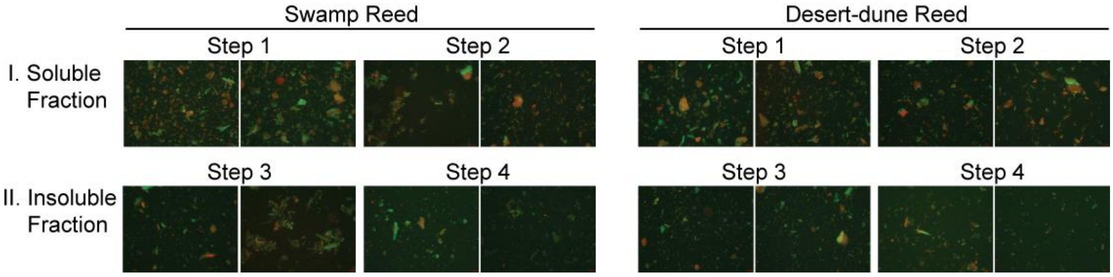

Figure S3. Remnants in the four-step extraction were checked using confocal microscopy to ensure consistency between ecotypes.

Supplementary Reference

1. Cui, S.; Hu, J.; Guo, S.; Wang, J.; Cheng, Y.; Dang, X.; Wu, L.; He, Y., Proteome analysis of *Physcomitrella patens* exposed to progressive dehydration and rehydration. *J Exp Bot* **2012,** 63, (2), 711-26.
2. Cui, S.; Hu, J.; Yang, B.; Shi, L.; Huang, F.; Tsai, S.; Ngai, S.; He, Y.; Zhang, J., Proteomic characterization of *Phragmites communis* in ecotypes of swamp and desert dune. *Proteomics* **2009,** 9, (16), 3950-67.
3. Liu, Y. G.; Mitsukawa, N.; Oosumi, T.; Whittier, R. F., Efficient isolation and mapping of Arabidopsis thaliana T-DNA insert junctions by thermal asymmetric interlaced PCR. *The Plant Journal* **1995,** 8, (3), 457-463.
4. Wu, L.; Di, D.-W.; Zhang, D.; Song, B.; Luo, P.; Guo, G.-Q., Frequent problems and their resolutions by using thermal asymmetric interlaced PCR (TAIL-PCR) to clone genes in Arabidopsis T-DNA tagged mutants. *Biotechnology & Biotechnological Equipment* **2015,** 29, (2), 260-267.
