# Supplementary material for: A Proteome Translocation Response to Complex Desert Stress Environments in Perennial Phragmites Sympatric Ecotypes with Contrasting Water Availability
Source: Front Plant Sci. 2017 Apr 13;8:511. doi: 10.3389/fpls.2017.00511 (PMC5390029; doi:10.3389/fpls.2017.00511)
Supplement: Supplementary file 2 [file Data_Sheet_2.pdf]

Li et al. A proteome translocation response to complex desert stress environments in perennial *Phragmites* sympatric ecotypes with contrasting water availability

Table S2. Identification of the translocation proteins in *Phragmites communis* using MALDI TOF/TOF MS.
Note: PM/SC, peptides matched/sequence coverage
Peptide Sequences, matched peptides from MS/MS spectra, with 95% confidence

Spot	Protein				Peptide Sequences		Mass (kDa)/pI		Species	Function catalogue	Function description
	Protein name	Accession No.	PM/SC	Protein Score	Sequence	Ions Score	Calc.	Meas.			
156-1	rubisco large subunit	gi|642404	18/33	424	TFQGPPHGIQVER	79	51.61/6.14	54.00/6.18	*Phragmites australis*	Energy	Photosynthesis
					DTDILAAFR	74					
					DNGLLLHIHR	73					
					EMTLGFVDLLR Oxidation (M)	52					
156-2	rubisco large subunit	gi|642404	19/41	407	TFQGPPHGIQVER	108	51.40/6.71	54.00/6.25	*Phragmites australis*	Energy	Photosynthesis
					DNGLLLHIHR	81					
					EMTLGFVDLLR Oxidation (M)	58					
156-3	rubisco large subunit	gi|642404	18/38	490	TFQGPPHGIQVER	95	51.40/6.71	54.00/6.30	*Phragmites australis*	Energy	Photosynthesis
					DTDILAAFR	79					
					DNGLLLHIHR	83					
					EMTLGFVDLLR Oxidation (M)	74					
156-4	rubisco large subunit	gi|642404	16/39	428	EMTLGFVDLLR	98	48.78/6.46	54.00/6.47	*Phragmites australis*	Energy	Photosynthesis
					TFQGPPHGIQVER	87					
					GGLDFTKDDENVNSQPFMR	52					
					EMTLGFVDLLR Oxidation (M)	41					
156-5	rubisco large subunit	gi|642404	20/42	388	TFQGPPHGIQVER	88	51.40/6.71	54.00/6.50	*Phragmites australis*	Energy	Photosynthesis
					DNGLLLHIHR	76					
					EMTLGFVDLLR Oxidation (M)	50					
156-6	rubisco large subunit	gi|642404	19/35	444	TFQGPPHGIQVER	92	51.60/6.23	54.00/6.52	*Phragmites australis*	Energy	Photosynthesis
					DNGLLLHIHR	67					
					DTDILAAFR	74					
					EMTLGFVDLLR Oxidation (M)	60					
21	rubisco large subunit (fragment)	gi|31087895	7/33	353	WSPELAAACEIWK Carbamidomethyl (C)	57	53.69/6.13	34.82/6.24	*Hordeum chilense*	Energy	Photosynthesis
					FVFCAEAIYK Carbamidomethyl (C)	53					
50	rubisco large subunit (fragment)	gi|9719179	10/25	154	EMTLGFVDLLR Oxidation (M)	33	51.625/6.33	16.11/5.19	*Proiphys cunninghamii*	Energy	Photosynthesis
					WSPELAAACEIWK Carbamidomethyl (C)	82					
348	rubisco large subunit (fragment)	gi|55792763	8/17	233	EITLGFVDLLR	59	51.61/6.04	15.41/5.28	*Uapaca bojeri*	Energy	Photosynthesis
					WSPELAAACEIWK Carbamidomethyl (C)	59					
350	rubisco large subunit (fragment)	gi|33636041	13/26	211	MSGGDHIHAGTVVGK	77	50.54/6.57	16.30/5.46	*Haworthiopsis glauca*	Energy	Photosynthesis
					EMTLGFVDLLR	73					
					WSPELAAACEIWK	65					
210	rubisco small subunit	gi|149392567	4/31	186	KAYPDAFVR	69	12.03/8.46	14.57/5.73	*Oryza sativa*	Energy	Photosynthesis
					LPMFGCTDATQVLK	98					
123	rubisco small subunit	gi|76574250	5/33	164	KFETLSYLPTLVDEVLLK	68	13.66/8.37	14.85/5.82	*Musa acuminata*	Energy	Photosynthesis

					SPGYYDGR	54					
120	rubisco small subunit	gi|76574250	6/40	160	SPGYYDGR	57	13.66/8.37	14.52/6.23	*Musa acuminate*	Energy	Photosynthesis
					KFETLSYLPTLVDEVLLK	41					
70	rubisco small subunit	gi|2407281	9/40	214	LPMFGCTDATQVLK	83	19.54/9.01	15.67/6.50	*Phragmites australis*	Energy	Photosynthesis
					LPMFGCTDATQVLK Carbamidomethyl (C)	68					
		gi|565824425		373	KFETLSYLPPLTTEDLLK	110					
					WIPCLEFSK	43					
					LPMFGCTDATQVLK	110					
					KAYPDAFVR	62					
414	ribulose activase	gi|12620883	10/29	394	GLAYDISDDQQDITR	72	48.32/5.06	46.36/5.53	*Gossypium hirsutum*	Energy	Photosynthesis
					VPIIVTGNDFSTLYAPLIR	68					
					LVDTFPGQSIDFFGALR	129					
					EGPPTFEQPK	50					
297	ribulose activase	gi|12620883	10/26	473	LVDTFPGQSIDFFGALR	115	48.32/5.06	47.19/5.48	*Gossypium hirsutum*	Energy	Photosynthesis
					GLAYDISDDQQDITR	106					
					VPIIVTGNDFSTLYAPLIR	83					
					VYDDEVR	40					
					EGPPTFEQPK	70					
298	ribulose activase	gi|7960277	11/30	379	GLAYDISDDQQDITR	120	47.78/6.92	45.83/5.41	*Triticum aestivum*	Energy	Photosynthesis
					VPIIVTGNDFSTLYAPLIR	70					
					LVDTFPGQSIDFFGALR	116					
396	ribulose activase	gi|12620883	10/27	367	GLAYDISDDQQDITR	106	48.32/5.06	49.27/5.5.26	*Gossypium hirsutum*	Energy	Photosynthesis
					VPIIVTGNDFSTLYAPLIR	41					
					LVDTFPGQSIDFFGALR	65					
					EGPPTFEQPK	70					
103	ribulose activase	gi|12620883	7/25	489	LVDTFPGQSIDFFGALR	127	48.32/5.06	46.00/5.43	*Gossypium hirsutum*	Energy	Photosynthesis
					MCALFINDLDAGAGR Carbamidomethyl (C)	93					
					MCALFINDLDAGAGR Carbamidomethyl (C) Oxidation	43					
					GLAYDISDDQQDITR	112					
					VPIIVTGNDFSTLYAPLIR	118					
304	ribulose activase	gi|12620883	10/30	473	GLAYDISDDQQDITR	73	48.32/5.06	46.00/5.48	*Gossypium hirsutum*	Energy	Photosynthesis
					SFQCELVFAK Carbamidomethyl (C)	49					
					LVDTFPGQSIDFFGALR	62					
					VPIIVTGNDFSTLYAPLIR	71					
109	ribulose activase	gi|415852	12/29	390	VPIIVTGNDFSTLYAPLIR	74	48.32/8.20	44.11/5.85	*Malus x domestica*	Energy	Photosynthesis
					SFQCELVFAK Carbamidomethyl (C)	42					
					LVDTFPGQSIDFFGALR	73					
176	ribulose activase	gi|3687652	10/30	268	LVDTFPGQSIDFFGALR	111	40.82/7.59	43.83/5.67	*Datisca glomerata*	Energy	Photosynthesis
					VPIIVTGNDFSTLYAPLIR	61					
175	ribulose activase	gi|7960277	12/36	307	LVDTFPGQSIDFFGALR	97	47.78/6.92	44.68/5.52	*Triticum aestivum*	Energy	Photosynthesis
					GLAYDISDDQQDITR	70					
					VPIIVTGNDFSTLYAPLIR	52					
320	ribulose activase	gi|12620883	10/	396	LVDTFPGQSIDFFGALR	74	48.32/5.06	42.79/6.07	*Gossypium hirsutum*	Energy	Photosynthesis

					VYDDEVR	40					
					VPLILGIWGGK	52					
					MCALFINDLDAGAGR Carbamidomethyl (C)	43					
					VPIIVTGNDFSTLYAPLIR	45					
318	glyceraldehyde-3-phosphate dehydrogenase	gi|50924788	10/36	500	YDSTLGIFDADVKPVGDNAISVDGK	83	42.69/7.62	42.96/5.90	*Oryza sativa*	Energy	Photosynthesis
					TLAEEVNQAFR	49					
					VVDLADIVANQWK	75					
					GTMTTTHSYTGDQR	72					
					VIAWYDNEWGYSQR	65					
					GILEVCDVPLVSVDFR	61					
					TCVLVSEEDEEATFVDPALR Carbamidomethyl (C)	50					
169	phosphoribulokinase	gi|50911777	13/47	281	IRDLYEQIIAER	67	44.84/5.68	45.25/5.24	*Oryza sativa*	Energy	Photosynthesis
					DLYEQIIAER	74					
420	phosphoribulokinase	gi|50911777	16/48	384	KPDFDAFIDPQK	89	44.84/5.68	46.06/5.28	*Oryza sativa*	Energy	Photosynthesis
					FYGEVTQQMLK	49					
					DLYEQIIAER	54					
149	ATP synthase alpha chain	gi|90110817	28/52	729	IIGLGEIMSGELVEFAEGTR Oxidation (M)	81	55.72/5.87	58.68/6.18	*Saccharum officinarum*	Transporters	Transport ATPases
					IAQIPVSEAYLGR	91					
					LIESPAPGIISR	89					
					EAYPGDVFYLHSR	129					
					QSQSNPLPVEEQVATIYTGTR	77					
146	ATP synthase alpha subunit	gi|49659509	27/52	729	IAQIPVSEAYLGR	75	55.66/5.87	59.86/6.00	*Saccharum officinarum*	Transporters	Transport ATPases
					LIESPAPGIISR	73					
					EAYPGDVFYLHSR	114					
					QSQSNPLPVEEQVATIYTGTR	63					
					GYLDSLEIEQVKK	99					
					EAIQEQLER	50					
166	ATP synthase beta subunit	gi|49659519	21/47	601	IFNVLGEPIDNLGPVDTSATFPIHR	61	53.93/5.31	48.16/5.41	*Saccharum officinarum*	Transporters	Transport ATPases
					AHGGVSVFGGVGER	138					
					FVQAGSEVSALLGR	102					
596	ATP synthase epsilon subunit	gi|227787	9/54	187	RIIWDCEVK	46	15.10/5.03	16.17/5.43	*Sorghum bicolor*	Transporters	Transport ATPases
					IRVEAVNWIPPSN	55					
567	40S ribosomal protein S16	gi|242084612	7/14	91	QAAAGGGDSLRVTETR	14	58.72/5.17	57.76/4.90	*Sorghum bicolor*	Protein synthesis	Ribosomal proteins
					SFPIQFPESFEQESLR	67					
592	50S ribosomal protein L10	gi|218192573	4/17	96	EQLEGCYLLAGIR	86	24.27/9.40	27.09/6.32	*Oryza sativa*	Protein synthesis	Ribosomal proteins
					QLQGIRDTLPETCRLLVAK	40					
600	50S ribosomal protein L31	gi|219362551	4/37	159	GLHPEIYEDAK	80	14.90/5.54	13.18/5.16	*Zea mays*	Protein synthesis	Ribosomal proteins
					EYWPEDQWR	41					
571	30S ribosomal protein S1	gi|357112501	7/14	134	FVEVDEEQGR	41	43.35/4.68	51.52/4.85	*Brachypodium distachyon*	Protein synthesis	Ribosomal proteins
					KLEPTPGDMIR	47					
206	translational elongation factor Tu	gi|50910077	11/32	496	KYDEIDAAPEER	63	50.38/6.19	50.10/5.42	*Oryza sativa*	Protein synthesis	Translation factors
					GITINTATVEYETETR	83					
					KDQVDDEELLQLVELEVR	104					

					MVVELIQPVACEQGMR	124					
					MVVELIQPVACEQGMR Carbamidomethyl (C)	57					
					MVVELIQPVACEQGMR Carbamidomethyl (C) Oxidatio	40					
573	translational elongation factor Tu	gi|50910077	9/26	268	KYDEIDAAPEER	89	50.38/6.19	50.48/5.36	*Oryza sativa*	Protein synthesis	Translation factors
					GITINTATVEYETETR	55					
					DQVDDEELLQLVELEVR	75					
205-1	chaperonin 60 α subunit	gi|113649074	17/37	477	LANAVGVTLGPR	42	61.09/5.12	60.86/5.16	*Oryza sativa*	Protein destination and storage	Folding and stability
					GYISPQFVTNLEK	62					
					AVLQDIAIVTGAEFLAK	141					
					AIELYDPMENAGAALIR	75					
					AIELYDPMENAGAALIR Oxidation (M)	51					
205-2	chaperonin 60 α subunit	gi|113649074	15/35	328	LANAVGVTLGPR	56	61.09/5.12	60.62/5.17	*Oryza sativa*	Protein destination and storage	Folding and stability
					AVLQDIAIVTGAEFLAK	89					
226	chaperonin 60 α subunit	gi|113649074	19/44	463	AVLQDIAIVTGAEFLAK	96	61.09/5.12	62.88/5.14	*Oryza sativa*	Protein destination and storage	Folding and stability
					ALVAPASLIAHNAGVEGEVVVEK	65					
262	chaperonin 60 α subunit	gi|50920285	9/21	169	AIELADPMENAGAALIR Oxidation (M)	69	61.36/5.36	60.24/5.28	*Oryza sativa*	Protein destination and storage	Folding and stability
					EIIPLLEQTTQLR	57					
207-1	chaperonin 60 β subunit	gi|34897924	16/38	519	LADLVGVTLGPK	80	64.05/5.60	60.70/5.38	*Oryza sativa*	Protein destination and storage	Folding and stability
					DLINVLEEAIR	56					
					NLIEAAEQEYEK	50					
					AAVEEGIVVGGGCTLLR Carbamidomethyl (C)	62					
					TNDLAGDGTTTSVVLAQGLIAEGVK	143					
207-2	chaperonin 60 β subunit	gi|34897924	17/40	486	LADLVGVTLGPK	75	64.05/5.60	60.86/5.43	*Oryza sativa*	Protein destination and storage	Folding and stability
					TNDLAGDGTTTSVVLAQGLIAEGVK	110					
					DLINVLEEAIR	44					
					NLIEAAEQEYEK	47					
					AAVEEGIVVGGGCTLLR	49					
207-3	chaperonin 60 β subunit	gi|34897924	9/27	259	DLINVLEEAIR	62	64.05/5.60	60.86/5.48	*Oryza sativa*	Protein destination and storage	Folding and stability
					AAVEEGIVVGGGCTLLR	41					
					TNDLAGDGTTTSVVLAQGLIAEGVK	70					
264-1	chaperonin 60 β subunit	gi|357110700	9/23	232	SSENNLYVVEGMQFDR	92	64.27/5.60	60.62/5.26	*Brachypodium distachyon*	Protein destination and storage	Folding and stability
					AAVEEGIVVGGGCTLLR	85					
264-2	chaperonin 60 β subunit	gi|357110700	14/31	277	AAVEEGIVVGGGCTLLR	100	64.27/5.60	60.86/5.31	*Brachypodium distachyon*	Protein destination and storage	Folding and stability
					SSENNLYVVEGMQFDR	129					
264-3	chaperonin 60 β subunit	gi|357110700	11/28	256	AAVEEGIVVGGGCTLLR	70	64.27/5.60	60.78/5.34	*Brachypodium distachyon*	Protein destination and storage	Folding and stability
					SSENNLYVVEGMQFDR	99					
261-1	chaperonin 60 β subunit	gi|222634842	12/33	170	TNDLAGDGTTTSVVLAQGLIAEGVK	43	59.03/5.15	58.83/5.16	*Oryza sativa*	Protein destination and storage	Folding and stability
					EVELEDPVENIGAK	56					
261-2	chaperonin 60 β subunit	gi|222634842	14/37	240	VVAAGANPVQITR	51	58.73/5.18	58.46/5.17	*Oryza sativa*	Protein destination and storage	Folding and stability
					TNDLAGDGTTTSVVLAQGLIAEGVK	49					
					EVELEDPVENIGAK	41					
564-1	chaperonin 60 β subunit	gi|357110700	13/23	161	SSENNLYVVEGMQFDR	104	63.87/5.60	60.16/5.20	*Brachypodium distachyon*	Protein destination and storage	Folding and stability
					AAVEEGIVVGGGCTLLR	40					

564-2	chaperonin 60 β subunit	gi|357110700	12/24	158	SSENNLYVVEGMQFDR	70	63.87/5.60	60.24/5.24	*Brachypodium distachyon*	Protein destination and storage	Folding and stability
					AAVEEGIVVGGGCTLLR	57					
131-1	70 kDa heat shock-like protein	gi|223974245	16/30	319	AVVTVPAYFNDSQR	81	64.93/5.51	68.24/4.98	*Zea mays*	Protein destination and storage	Folding and stability
					IPAVQELVR	61					
					SEVFSTAADGQTSVEINVLQGER	129					
131-2	70 kDa heat shock-like protein	gi|223974245	9/21	139	AVVTVPAYFNDSQR	71	64.97/5.52	68.32/5.00	*Zea mays*	Protein destination and storage	Folding and stability
					IPAVQELVR	45					
131-3	70 kDa heat shock-like protein	gi|7269278	15/22	366	QFAAEEISAQVLR	107	76.51/5.07	68.24/5.02	*Arabidopsis thaliana*	Protein destination and storage	Folding and stability
					IPAVQELVR	68					
					SEVFSTAADGQTSVEINVLQGER	73					
131-4	70 kDa heat shock-like protein	gi|223974245	17/22	112	AVVTVPAYFNDSQR	56	64.93/5.51	67.72/5.04	*Zea mays*	Protein destination and storage	Folding and stability
					IPAVQELVR	42					
108	heat shock protein 70	gi|113648993	12/19	335	FEELCSDLIDR Carbamidomethyl (C)	45	74.04/5.11	68.24/5.13	*Oryza sativa*	Protein destination and storage	Folding and stability
					QFAAEEISAQVLR	66					
					IINEPTAASLAYGFEK	95					
					SEVFSTAADGQTSVEINVLQGER	47					
560	90 kDa heat shock-like protein	gi|357158923	21/28	120	VFISDDFDGELFPR	55	89.49/5.11	73.18/5.09	*Brachypodium distachyon*	Protein destination and storage	Folding and stability
					TVEVEEEEESK	42					
580	Peptidyl-prolyl cis-trans isomerase	gi|115476198	17/44	300	IKDNPNLEDCVFR	84	46.45/4.82	46.47/4.91	*Oryza sativa*	Protein destination and storage	Folding and stability
					FYDGMEIQR	41					
					APVYGETLEELGR	106					
562	endosperm lumenal binding protein	gi|2267006	20/33	164	FEELNNDLFR	45	73.50/5.30	67.03/5.32	*Oryza sativa*	Protein destination and storage	Folding and stability
					ALSNQHQVR	41					
214	ATP-binding subunit of ATP-dependent Clp protease	gi|50923949	31/46	472	GSGFVAVEIPFTPR	44	98.44/5.79	73.83/5.72	*Oryza sativa*	Protein destination and storage	Proteolysis
					QLGHNYIGSEHLLLGLLR	64					
					VPEPTVDETIQILR	74					
239	ATP-dependent Clp protease,ATP-binding subunit	gi|50923949	23/33	403	GSGFVAVEIPFTPR	52	98.44/5.79	74.30/5.77	*Oryza sativa*	Protein destination and storage	Proteolysis
					VPEPTVDETIQILR	77					
					LIGSPPGYVGYTEGGQLTEAVR	64					
472	2-Cystenin peroxiredoxin	gi|296514188	10/39	352	EGVIQHSTINNLAIGR	109	28.10/5.67	26.83/4.93	*Oryza sativa*	Disease/defence	Stress responses
					SGGLGDLKYPLISDVTK	88					
					SFGVLIPDQGIALR	56					
361	2-Cystenin peroxiredoxin	gi|11353651	6/31	261	YPLISDVTK	55	28.08/5.67	26.95/5.02	*Secale cereale*	Disease/defence	Stress responses
					SFGVLIPDQGIALR	77					
					EGVIQHSTINNLAIGR	65					
113	2-Cystenin peroxiredoxin	gi|3328221	5/40	277	APDFAAEAVFDQEFINVK	116	28.12/6.34	26.95/5.09	*Oryza sativa*	Disease/defence	Stress responses
					SFGVLIPDQGIALR	64					
					TLQALQYVQENPDEVCPAGWKPGEK	56					
591	2-Cystenin peroxiredoxin	gi|116310804	5/38	167	EGVIQHSTINNLAIGR	72	18.11/4.67	26.02/5.11	*Oryza sativa*	Disease/defence	Stress responses
					SFGVLIPDQGIALR	45					
602	peroxiredoxin-2E-2	gi|115444771	10/40	174	LPDATLSYFDPADGELK	90	23.17/6.15	18.02/5.02	*Oryza sativa*	Disease/defence	Stress responses
					ALGVEMDLSDKPMGLGVR	68					
597	thioredoxin M	gi|4138290	9/26	136	MIAPVIDELAK Oxidation (M)	70	18.53/8.16	15.00/5.42	*Oryza sativa*	Disease/defence	Stress responses

					TTLATIIDK	42					
132	plastidic glutamine synthetase	gi|44885918	17/36	363	HDLHISAYGEGNER	119	46.74/6.22	48.0/5.48	*Phragmites australis*	Disease/defence	Stress responses
					LTGLHETASIDNFSWGVANR	94					
574	plastidic glutamine synthetase	gi|44885918	6/19	112	HDLHISAYGEGNER	43	46.74/6.22	48.59/5.31	*Phragmites australis*	Disease/defence	Stress responses
					LTGLHETASIDNFSWGVANR	46					
185	14-3-3-like protein	gi|37903393	10/49	392	TVDVEELTVEER	105	28.86/4.79	31.94/5.11	*Saccharum hybrid cultivar*	Unclear classification	Unclear classification
					AAQDIALAELAPTHPIR	115					
					QAFDEAISELDTLGEESYK	66					
579	adenosine kinase-like protein	gi|125540364	8/20	185	RIAVITQGADPVVVAEDGQVK	52	34.24/5.02	47.37/5.22	*Oryza sativa*	Unclear classification	Unclear classification
					HLPMYDELASK	49					
7	mRNA binding protein	gi|26453355	5/11	103	DCEEWFFDR	41	43.91/7.10	42.84/6.10	*Lycopersicon esculentum*	Unclear classification	Unclear classification
					FSEITGAGGR	46					
598	RNA-binding protein	gi|2226370	4/32	114	EGGYGGGGGGYGGGR	45	15.74/5.56	14.24/5.41	*Nicotiana glutinosa*	Unclear classification	Unclear classification
					GFGFVTFKDEQAMR	40					
