# Supplementary material for: A Proteome Translocation Response to Complex Desert Stress Environments in Perennial Phragmites Sympatric Ecotypes with Contrasting Water Availability
Source: Front Plant Sci. 2017 Apr 13;8:511. doi: 10.3389/fpls.2017.00511 (PMC5390029; doi:10.3389/fpls.2017.00511)
Supplement: Supplementary file 3 [file Data_Sheet_3.pdf]

Table S5. Unknown proteins that translocated to the insoluble fraction of DR.

No	Spots No.	Fractions in SR			Fractions in DR			Translocation rate
		Sol.%	Inso. %	*P* value	Sol. %	Inso. %	*P* value	DR_{inso}- SR_{inso} (%)
1	565	55.9	44.1	0.028	15.5	84.5	0.001	40.5
2	566	56.7	43.3	0.010	20.0	80.0	0.017	36.7
3	568	100.0	0.0	0.009	20.1	79.9	0.035	79.9
4	569	61.4	38.6	0.028	14.0	86.0	0.003	47.4
5	570	52.6	47.4	0.043	6.6	93.4	0.006	46.0
6	572	28.7	71.3	0.003	3.0	97.0	0.001	25.8
7	575	49.8	50.2	0.001	12.2	87.8	0.006	37.6
8	59	65.6	34.4	0.028	35.7	64.3	0.008	29.9
9	578	64.4	35.6	0.002	33.2	66.8	0.013	31.2
10	582	100.0	0.0	0.004	36.6	63.4	0.048	63.4
11	583	69.8	30.2	0.004	22.1	77.9	0.013	47.7
12	584	47.6	52.4	0.003	13.8	86.2	0.004	33.8
13	588	52.6	47.4	0.049	20.0	80.0	0.014	32.6
14	590	28.9	71.1	0.007	4.2	95.8	0.006	24.8
15	593	35.1	64.9	0.045	6.4	93.6	0.009	28.7
16	594	33.8	66.2	0.007	7.4	92.6	0.004	26.3
17	595	29.7	70.3	0.022	4.5	95.5	0.001	25.2
18	599	56.5	43.5	0.041	17.8	82.2	0.002	45.7
19	601	55.8	44.2	0.011	30.3	69.7	0.004	25.4

P value (<0.05) shows T-test of sol/inso ratio for each protein spot, from built-in support of DeCyder software v6.5
